# Molecular biogeography of planktonic and benthic diatoms in the Yangtze River

**DOI:** 10.1186/s40168-019-0771-x

**Published:** 2019-12-05

**Authors:** Jiawen Wang, Qingxiang Liu, Xianfu Zhao, Alistair G. L. Borthwick, Yuxin Liu, Qian Chen, Jinren Ni

**Affiliations:** 1 College of Environmental Sciences and Engineering, Key Laboratory of Water and Sediment Sciences, Ministry of Education, Peking University, Beijing, 100871 China; 2 Institute of Hydroecology, Ministry of Water Resources, Chinese Academy of Sciences, Wuhan, 430079 China; 3 Institute of Infrastructure and Environment, School of Engineering, University of Edinburgh, The King’s Buildings, Edinburgh, EH9 3JL UK; 4 State Key Laboratory of Plateau Ecology and Agriculture, Qinghai University, Xining, 810016 China; 5 Beijing Innovation Center for Engineering Science and Advanced Technology, Peking University, Beijing, 100871 China

**Keywords:** Diatoms, Biogeography, Water, Sediment, Landform, Environmental driver, Human interference, Yangtze River

## Abstract

**Background:**

Diatoms are of great significance to primary productivity in oceans, yet little is known about their biogeographic distribution in oligotrophic rivers.

**Results:**

With the help of metabarcoding analysis of 279 samples from the Yangtze River, we provided the first integral biogeographic pattern of planktonic and benthic diatoms over a 6030 km continuum along the world’s third largest river. Our study revealed spatial dissimilarity of diatoms under varying landforms, including plateau, mountain, foothill, basin, foothill-mountain, and plain regions, from the river source to the estuary. Environmental drivers of diatom communities were interpreted in terms of photosynthetically active radiation, temperature, channel slope and nutrients, and human interference. Typical benthic diatoms, such as *Pinnularia*, *Paralia*, and *Aulacoseira*, experienced considerable reduction in relative abundance downstream of the Three Gorges Dam and the Xiluodu Dam, two of the world’s largest dams.

**Conclusions:**

Our study revealed that benthic diatoms are of particular significance in characterizing motile guild in riverine environments, which provides insights into diatom biogeography and biogeochemical cycles in large river ecosystems.

## Background

Diatoms play a particularly important role in the biogeochemical cycle [1] of primary elements such as carbon, nitrogen, phosphorus, and silica, contributing about 20–25% of global primary production [2]. Diatoms are ubiquitous and diverse species of single-celled, eukaryotic, photosynthetic microorganisms [3], and are often the dominant primary producers in marine and freshwater ecosystems [4]. Therefore, diatoms in such ecosystems may be remarkably dissimilar either in phylogenetic composition or biogeographic distribution [5, 6]. Freshwater bodies typically consist of lentic (particularly lakes and wetlands) and lotic waters (including streams and rivers), which are often dominated respectively by planktonic algae and benthic species [7].

Accurate identification of diatoms depends on the reliability of the methods used. Morphological analysis requires extensive taxonomic expertise and may exhibit shortcomings in characterizing specific diatoms in rivers [8]. With the development of high-throughput sequencing (HTS) technology, DNA metabarcoding has become a rapid, accurate, and reliable method for diatom detection [9]. Various DNA barcoding studies have been successfully conducted, based on different maker genes, including COI [10], ITS [11], and 18S rDNA [9, 12]. Malviya et al. [13] provided a new estimate of diversity and distribution of marine planktonic diatoms based on the V9 region of eukaryotic 18S rDNA. As a result, the most widespread and diverse diatom genera are derived from 46 marine stations. Recently, the V4 region of 18S rDNA was proposed for diatom barcoding in studies of diatoms in river and deltaic systems [9, 12].

Comparing the numerous studies of diatoms and eutrophication in oceans [14, 15] and lakes [16, 17] to date, it is clear that the present understanding of diatoms is relatively poor for lotic and oligotrophic rivers [7]. In fact, previous reports on the dynamics of riverine diatoms have mostly focused on tributaries, small rivers, reaches, stations, and estuaries [18–20]. Many studies examined the diversity and composition of planktonic [19, 20] or benthic diatoms [18, 21, 22] based on morphological identification. For example, Centis et al. [20] investigated planktonic diatoms dominated by physical constraints at two stations of the River Adige, Italy. Liu et al. [18] investigated the community structure of benthic diatoms in the Dong River, one of the three main tributaries of the Pearl River, China. Although Kireta et al. [23] observed that both planktonic and periphytic diatoms could be used as bio-indicators of river conditions, little is known about the distinction between planktonic and benthic diatoms regarding their spatiotemporal distributions.

Biogeography studies aim to reveal the spatial and temporal distribution of biodiversity and provide insight into the mechanisms that generate and sustain diversity [24]. Spatial dispersal and environmental selection processes are regarded as essential drivers for the biogeographical pattern of bacterial community [25]. The former promotes movement of species and their establishment at a new location, whereas the latter alters the abundance and composition of species, according to the ability to survive and reproduce under local environmental conditions. A similar explanation has been proved to apply to the biogeographical pattern of planktonic or benthic diatom communities in small rivers using morphological analysis [26–28]. However, it remains unclear how the integrated spatiotemporal distributions of planktonic and benthic diatom communities are shaped by spatial dispersal and environmental selection processes in large rivers subject to complex natural and anthropogenic impacts.

**Fig. 1:**
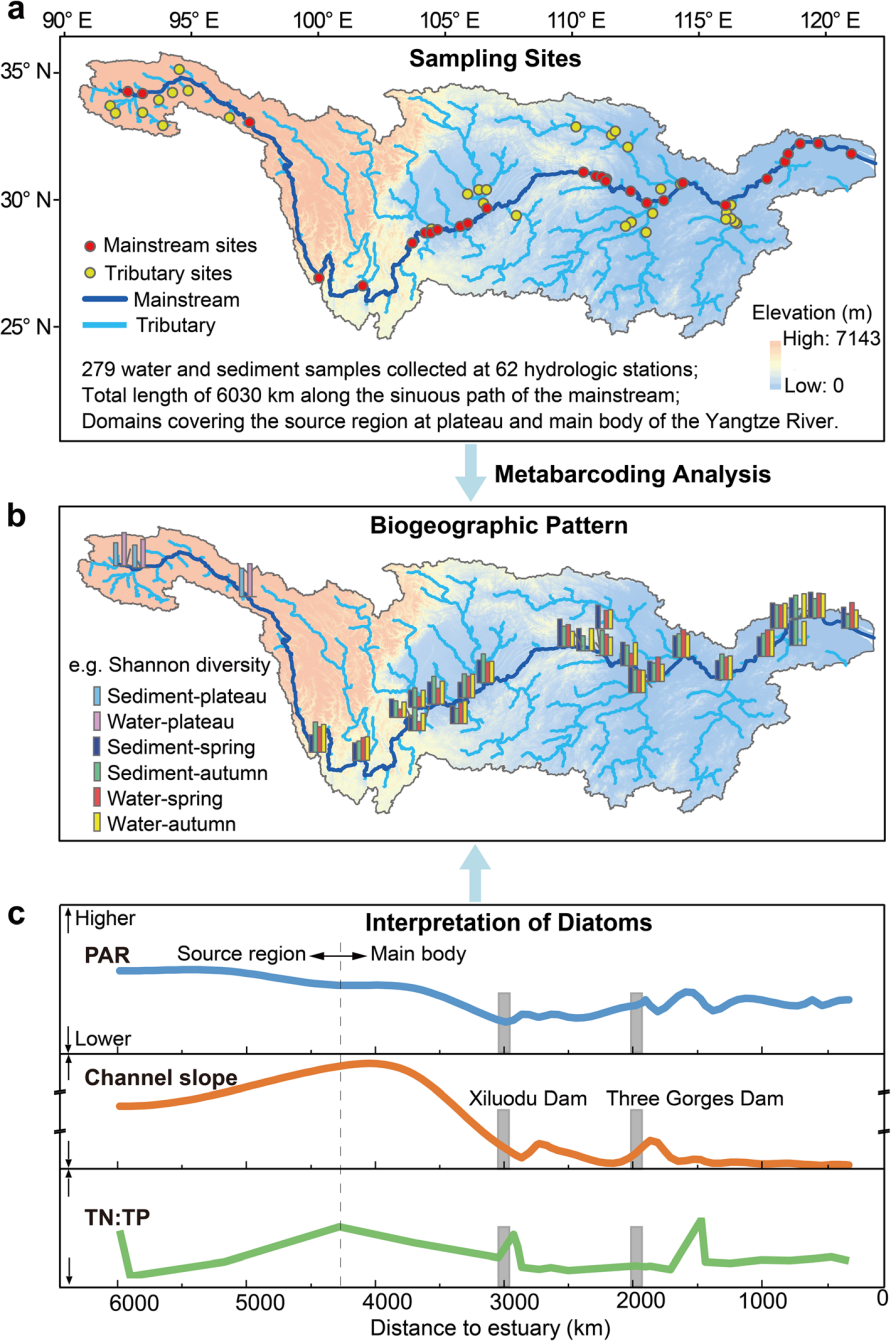
Flowchart of the study. **a** Two hundred seventy-nine water and sediment samples at 62 hydrologic stations in the Yangtze River covering the actual sinuous channel reach of length 6030 km (equivalent to 1.83 times the 3290 km straight line from start to end sampling sites). **b** Metabarcoding analysis provides insights into biogeographic pattern of diatoms along the mainstream of the Yangtze River, represented by the spatial distribution of Shannon diversity. **c** Interpretations on the biogeographic patterns of diatom communities, with main influencing factors such as photosynthetically active radiation (PAR), channel slope, and nutrients characterized by ratio of total nitrogen to total phosphate (TN:TP)

To close the above gap, we implemented large-scaled synchronous monitoring of diatom communities at 62 hydrologic stations over a 6030 km continuum of the Yangtze River in China. Consequently, we provided the first molecular biogeographic pattern of both planktonic and benthic diatoms in the largest river in Asia (Fig. 1). Meanwhile, environmental drivers of diatom communities were interpreted in terms of photosynthetic active radiation, temperature, channel slope, and nutrient conditions under varying landforms.

## Results

Our study generated a total of 8,602,620 V4 18S rDNA reads from 279 samples. All sequencing reads were classified into 3947 operational taxonomic units (OTUs) at a 97% similarity threshold, with 3144 OTUs well matching 454 diatom species in our reference database. Rarefaction curves (Additional file 1: Figure S1) together with high values of Good’s coverage ranging from 0.9854 to 0.9992 illustrated that OTUs obtained by the current sequencing depth gave a reasonable representation of the diatom communities. The phylogeny tree, constructed by representative OTUs (accounting for > 90% sequence in all samples) and reference sequences (Additional file 1: Figure S2), further confirmed the accuracy of taxonomic assignment.

### Alpha and beta diversity of diatom communities

Molecular barcoding based on high-throughput sequencing (HTS) provided a detailed diatom directory for the whole Yangtze River at different taxonomy levels, i.e., 4 classes, 37 orders, 60 families, and 152 genera.

HTS is of particular use in detecting nano-sized diatoms (2–20 μm) in the Yangtze River, confirming the presence of *Fragilaria perminuta*, *Achnanthidium minutissimum*, *Achnanthidium saprophilum*, *Amphora pediculus*, *Fistulifera saprophila*, *Mayamaea permitis*, *Sellaphora seminulum*, *Encyonema minutum*, *Fragilaria famelica*, *Fragilaria rumpens*, *Gomphonema pumilum*, *Staurosirella pinnata*, *Planothidium frequentissimum*, *Craticula buderi*, *and Craticula molestiformis*.

Six types of environmental samples were taken along the Yangtze River, including water and sediment samples from the river source region (i.e., water-plateau (12 samples) and sediment-plateau (12 samples)) and those from the mainstream in the non-plateau area (i.e., water-spring (38 samples), water-autumn (46 samples), sediment-spring (87 samples), and sediment-autumn (84 samples)). Planktonic diatoms exhibited the highest alpha-diversity (Chao1 and Shannon indices) and benthic diatoms the lowest richness (Chao1) in the plateau (Additional file 1: Figure S3). In the non-plateau area, no significant differences were observed in the alpha richness and diversity of diatom communities in the four sample types.

Non-metric multidimensional scaling (NMDS) analysis of the compositional dissimilarities between diatom communities demonstrated not only a clear spatial differentiation in diatoms between the plateau and the main body of the Yangtze, but also a division between planktonic and benthic groups (Additional file 1: Figure S4). Water and sediment samples between spring and autumn in the non-plateau area were used for further seasonal analysis. Seasonal difference in planktonic diatoms was found much more significant than in benthic diatoms, as further confirmed by an analysis of similarity (ANOSIM) test (Additional file 1: Figure S5). Moreover, one-way analysis of variance (one-way ANOVA) indicated that more planktonic diatoms (42.75 ± 13.98% relative abundance, primarily belonging to *Cyclotella*, *Stephanodiscus*, and *Skeletonema*) than benthic diatoms (16.58 ± 5.06% relative abundance, primarily belonging to *Pinnularia* and *Stephanodiscus*) exhibited significant seasonal sensitivity (Additional file 1: Figure S6).

### Biogeographic patterns of diatom communities

A variety of diatom species have been found closely relevant to carbon export [29]. In the Yangtze River, the planktonic diatoms such as *Asterionella formosa*, *Diatoma vulgare*, *Lindavia viaradiosa*, *Gomphonema pumilum*, *and Thalassiosira nordenskioeldii* were significantly strongly associated with dissolved carbon dioxide (*p*CO_2_, see the “Methods” section) (Spearman *r* > 0.3, *P* < 0.05), while the benthic diatoms *Asterionella formosa*, *Encyonema prostratum*, *Eucocconeis laevis*, *Fistulifera saprophila*, and *Nitzschia sigmoidea* were highly correlated with *p*CO_2_ (Additional file 1: Figure S7).

**Fig. 2:**
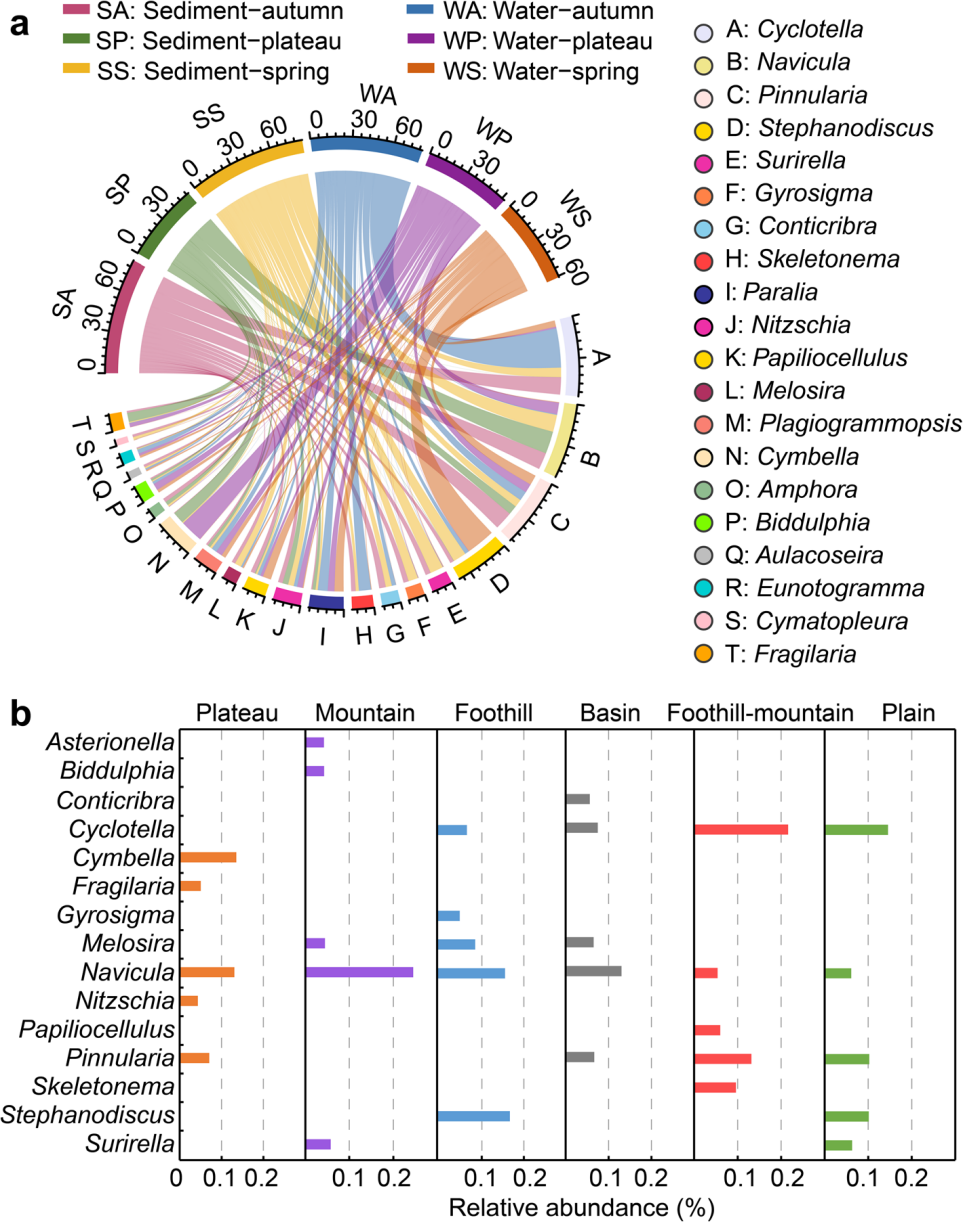
**a** Circular visualization of dominant diatoms at genus level in six sample types. Inner circular diagram shows relative abundance of different diatom genus in six sample types. Only the dominant genus with a mean relative abundance of ≥ 1% in all samples is depicted. The width of ribbons for each diatoms is directly proportional to their relative abundance in each sample type. Similarly, different colored ribbons of different width for each sample type describe the distribution of different genera. **b** Representative diatoms genera in different landform types from the river source to mouth along the Yangtze River

Obvious difference in species composition was observed in planktonic and benthic diatoms. In the Yangtze River, diatoms mainly consisted of *Coscinodiscophyceae*, *Fragilariophyceae*, *Bacillariophyceae*, and *Mediophycea*. Planktonic diatoms were dominated by *Coscinodiscophyceae* (about 43.76% of the total number of sequences) and *Mediophyceae* (17.91%), while benthic diatoms were dominated by *Bacillariophyceae* (54.88%) and *Coscinodiscophyceae* (30.96%) (Additional file 1: Figure S8). Planktonic and benthic diatoms were not always consistent in dominant genera (top 20, the relative abundance ranged from 55.6 to 83.6%) (Fig. 2a). The dominant genera were found to be *Cyclotella*, *Stephanodiscus*, *Pinnularia*, and *Paralia*, represented 12.2, 8.6, 7.3, and 6.6% of total sequences, respectively, in water samples. Meanwhile, *Navicula*, *Pinnularia*, and *Cyclotella* became the dominant genera, represented 14.4, 9.1, and 6.9% of total sequences, respectively, in sediment samples, in which *Navicula* was dominant in either sediment-plateau (17.1%), sediment-autumn (13.1%), or sediment-spring samples (13.2%).

Over the 6030 km continuum from river source to mouth, landform played a significant role in spatial differentiation of both planktonic and benthic diatom communities. Referring to a previous study on landform types in the Yangtze [30], planktonic diatoms were represented by *Cymbella*, *Asterionella*, *Stephanodiscus*, *Melosira*, *Cyclotella*, *and Conticribra* in the plateau, mountain, foothill, basin, foothill-mountain, and plain regions, respectively, while benthic diatoms were abundant by *Cymbella*, *Navicula*, *Melosira*, *Conticribra*, *Cyclotella*, and *Surirella*, respectively, in the corresponding regions (Fig. 2b).

A completed description on biogeographic pattern over a large river requires to identify the difference in diatom compositions among different types of samples and their spatiotemporal heterogeneity. Using the indicator species analysis, the diatoms that were responsible for the observed community differences among the six types of samples could be well identified (Additional file 1: Table S1). The number of indicator diatom species in the river ranged from 6 (sediment-spring) to 41 (water-plateau). Diatom communities in the plateau region were quite different from those in the non-plateau region of the Yangtze River, as evidenced by the higher percentage of top indicator species in water-plateau and sediment-plateau samples (Additional file 1: Figure S9). The average relative abundance of indicator species in the source area exceeded 40%, and planktonic indicator species contributed more reads than benthic indicator species. Furthermore, a number of indicator species belonging to *Tabellariales* and *Hemiaulales* occurred in water-plateau and sediment-plateau samples, respectively.

Diatom composition in terms of ecological guilds showed spatial dissimilarity in water and sediment samples (Additional file 1: Figure S10). Diatoms were divided into four ecological guilds according to their biological traits, including low-profile, high-profile, motile, and planktic guilds in terms of different responses to nutrients and dynamic disturbances [31–33] (see “Ecological guilds classification” section). Benthic diatoms in the motile guild prevailed at most stations along the whole river, whereas those in high-profile and planktic guilds dominated upstream and downstream reaches, respectively. In addition, planktonic diatoms in the planktic guild were predominant at most stations along the Yangtze River.

### Environmental effects on diatom biogeography

Significant distance-decay in diatom similarity was observed along the geographical distance (Additional file 1: Figure S11), with a greater slope of the curve for water (slope = − 0.042) than for sediment (slope = − 0.038) using least squares linear regression. The partial Mantel test demonstrated that both geographical and environmental distances played important roles in constraining diatom composition and distribution (Additional file 1: Table S2). Canonical correspondence analysis (CCA) showed significant correlations between diatom communities and specific environmental and spatial factors such as water temperature, pH, suspended solids, and PCNM-1 (Additional file 1: Table S3). Variation partitioning of diatom composition showed that a greater percentage (14.6–21.2%) could be explained by a purely environmental component than that (3.4–6.0%) of the total variation by a pure spatial component (Additional file 1: Figure S12), and a minor portion (0.4–5.4%) explained by spatially structured environmental heterogeneity, leaving the majority of the total variation (68.7–79.0%) inexplicable. As a deterministic process, environmental selection played a critical role in the biogeography of planktonic and benthic diatoms. Although environmental differentiation seemed more important than spatial dispersing in shaping a diatom community, neither could fully explain the total variation in diatom composition. Among others, the typical environmental components such as photosynthetically active radiation, temperature, channel slope, and nutrients condition are essential to diatom community accompanied with the spatial dispersal.

**Fig. 3:**
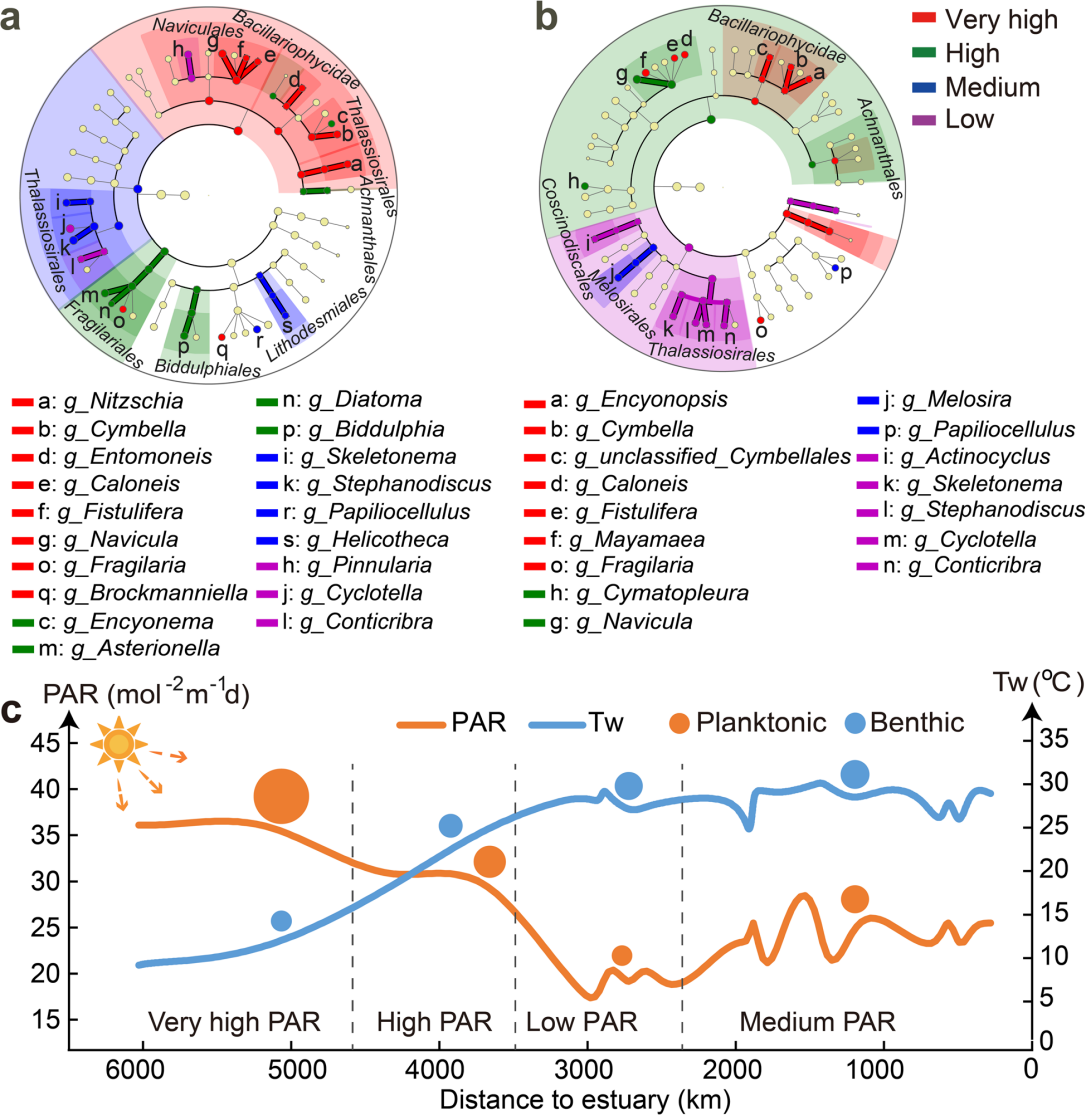
LEfSe cladogram of planktonic (**a**) and benthic (**b**) diatom communities from four PAR regions. Diatom taxa with a mean relative abundance of ≥ 0.1% in all samples, assigned to kingdom (innermost), phylum, class, order, family, and genus (outermost), are used to determine taxa or clades most likely to explain differences between PAR regions. Differentially abundant taxa (biomarkers) are colored by their most abundant PAR regions, i.e., red, green, blue, and purple circles stand for biomarkers in regions of very high, high, medium, and low. Orange and blue circles display the average alpha-diversity (Chao1) of planktonic and benthic diatoms respectively in different photosynthetically active radiation (PAR) regions, and their sizes correspond to the Chao1 index (**c**)

Photosynthetically active radiation (PAR, 400–700 nm) is utilized by diatoms to synthesize biomass through photosynthesis [34]. Spatially, the annual-averaged PAR exhibits four stages along the Yangtze River [35], i.e., the highest in the upper reach located in Qinghai-Tibet Plateau region (above 32 mol m^−2^ d^−1^), the higher in the reach located in the Hengduan Mountains, the lowest in the reach located in the Sichuan Basin (below 23 mol m^−^ d^−1^), and the moderate in the lower reach (Fig. 3c, see the “Photosynthetically active radiation (PAR) divisions” section). For a better understanding of the spatial heterogeneity of both planktonic and benthic diatom communities, LefSe analysis was used considering its advantages in identifying differentially abundant taxa under different environmental conditions [36]. Consequently, preferred planktonic and benthic diatom species in different PAR regions were identified (Fig. 3a, b). For example, the *Caloneis*, *Cymbella*, *Fistulifera*, and *Fragilaria* genera preferred very-high PAR zones, the *Papiliocellulus* genus favored medium PAR regions, and *Conticribra* and *Cyclotella* showed a preference for low PAR habitats. Planktonic *Cymatopleura* and *Navicula*, and benthic *Asterionella*, *Biddulphia*, *Diatoma*, and *Encyonema* genera preferred to high PAR conditions. Moreover, water temperature is a key environmental factor in structuring diatom community assemblages through its influence on diatom size and growth rate [37] in the Yangtze River (Additional file 1: Table S3). Although the richness of planktonic diatoms seemed to fluctuate with PAR, the richness of benthic diatoms tended to rise with increasing temperature (Fig. 3c).

**Fig. 4:**
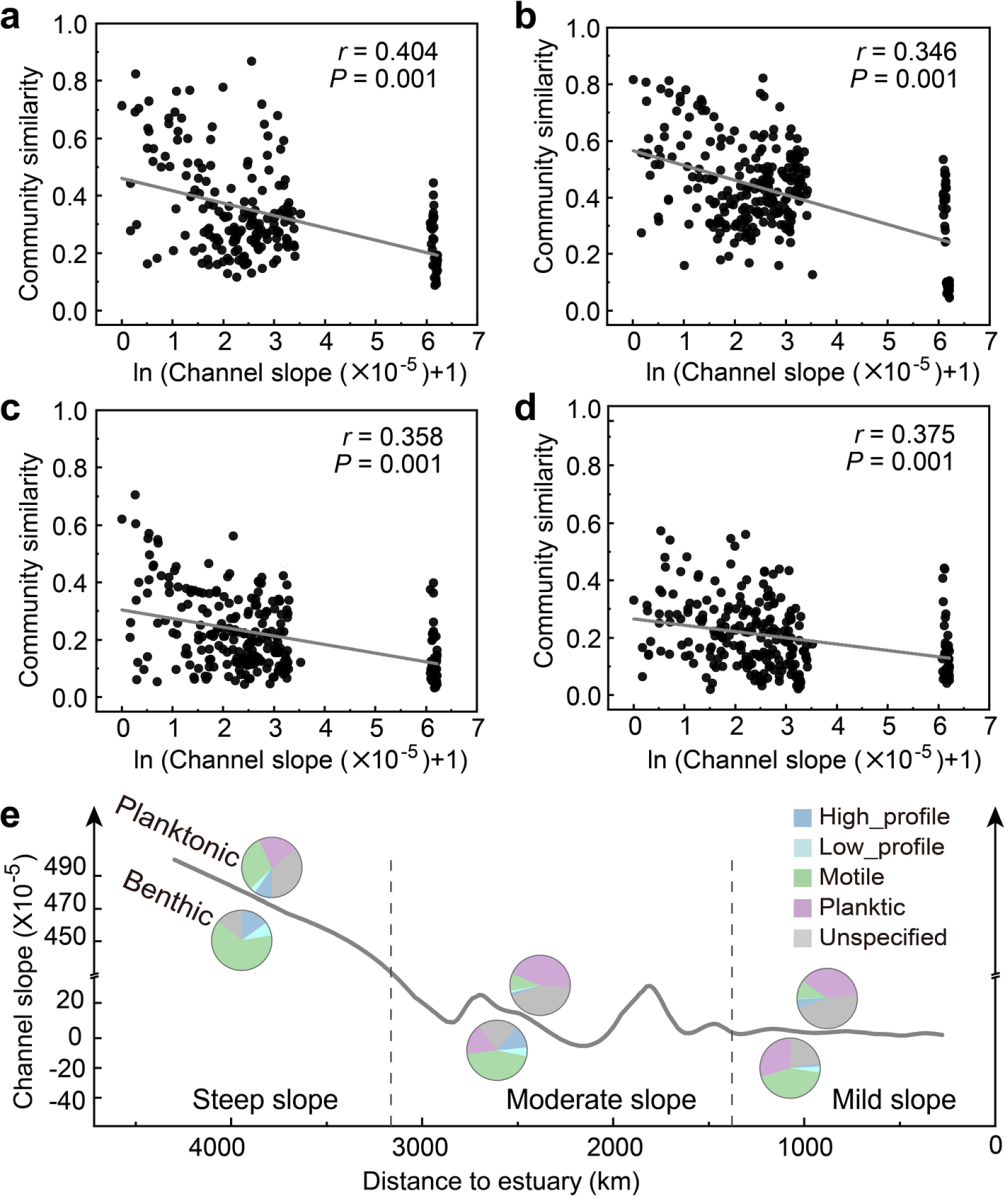
Relationships between community similarity and river channel slope for water-spring (**a**), water-autumn (**b**), sediment-spring (**c**), and sediment-autumn (**d**) samples. Values of Mantel Spearman correlations (*r*) and probabilities (*P*) are also provided. Gray lines denote ordinary least squares linear regression fits across all samples. Spatial distributions of ecological guilds for different channel slopes are shown in (**e**)

Stream power, often simply characterized by the river channel slope or the product of channel slope and flow discharge (except in plateau regions) [38], is another important factor altering the spatial distribution of diatoms. The channel slope dramatically changes along the Yangtze River, primarily due to the basis of geology, climate and geomorphology. In the mountainous reaches (stations 1~2), the river channel slope can be higher than 400 × 10^−5^. In the upper reaches (stations 3~14), the channel slope has dropped sharply to 10–30 × 10^−5^. In middle-lower reaches (stations 15~24), the slope of the riverbed is nearly to zero as the channel widens and shallows in the estuarine region. In general, the varying channel slope along the Yangtze River could be simplified into three stages, steep slope in mountainous reaches, moderate slope in upper reaches, and mild slope in middle-lower reaches (Fig. 4c). During the wet season (autumn), the higher flow discharge weakens the correlation between planktonic community similarity and channel slope, although a stronger correlation between benthic community similarity and channel slope is maintained due to higher mobility of the streambed (Fig. 4a, b). In view of their relative abundance, planktonic diatoms were characterized by *Psammothidium*, *Nitzschia*, and *Cymbella* for steep slope environments, *Papiliocellulus* for moderate slope, *Mayamaea*, *Pinnularia*, and *Surirella* for mild slope environments. Benthic diatoms were represented by *Cocconeis*, *Entomoneis*, and *Melosira* for steep slope environments, *Fallacia*, *Psammothidium*, and *Skeletonema* for moderate slopes, and *Actinocyclus*, *Aulacoseira*, and *Conticribra* for mild slopes (Additional file 1: Figure S13). Furthermore, slope effects on diatoms might be identified in terms of ecological guilds. Regardless of the diatoms in an unspecified ecological guild, planktonic diatoms were dominant in the planktic guild. Interestingly, species in motile guild stably constituted the main component of benthic diatoms in the whole lotic river (Fig. 4c).

Nutrient condition, represented by the total nitrogen to total phosphorus ratio (TN:TP), was considered as an indicator of ecosystem to support for algae biomass [39]. Since diatoms were reported to predominate in phytoplankton at high nitrogen to phosphorus ratio (N:P > 16) in water [40], we investigated the response of planktonic and benthic diatoms to TN:TP in the Yangtze River. Higher TN:TP was observed in water samples (13.8~45.63) than in sediment samples (0.004~0.65). The spatial distribution of TN:TP in water and sediment samples varied in different sampling stations. The lowest value of TN:TP in water samples was obtained in river reaches receiving large inflow from the Min River, and the highest value was obtained in both water and sediment samples near river estuary. Moreover, TN:TP exhibited a fluctuation between upstream and downstream of large dams (Xiluodu Dam and Three Gorges Dam). The alpha-diversity of planktonic diatoms could be partly interpreted in terms of annual-averaged dissolved TN:TP (data range from 2005 to 2014) in water-spring (Adj *R*^2^ = 0.54) and water-autumn (Adj *R*^2^ = 0.41) samples (Additional file 1: Figure S14a-b); however, benthic diatoms demonstrated an even weaker response to TN:TP (monitored data) (Additional file 1: Figure S14c-d). Furthermore, the monitored TN:TP was significantly related to community composition of diatoms in both water and sediment (*P* < 0.05, Additional file 1: Table S3). For relatively oligotrophic rivers like the Yangtze River, it appeared that TN was more important than TP as limiting nutrients to planktonic and benthic community variability (Additional file 1: Table S3).

Nevertheless, the aforementioned environmental factors are subject to change due to human interference. For the Yangtze River, one of the most prominent impacts on its ecology arises from the construction and operation of large dams. In the present study, one-way ANOVA analysis revealed that certain OTUs exhibited significant variations in distribution immediately upstream and downstream of the Three Gorges Dam (*P* < 0.01) and Xiluodu Dam (*P* < 0.05). Sudden drops in relative abundance of OTUs belonging to specific benthic species (such as *Pinnularia*, *Paralia*, and *Aulacoseira*) occurred downstream of the dams (Additional file 1: Figure S15). Furthermore, one-way ANOVA analysis also revealed significant difference in environmental variables (pH (*P* = 0.003), NH_4_-N (*P* < 0.001), TN (*P* < 0.001), and TN:TP (*P* = 0.01)) for sediment samples immediately before and after the Three Gorges Dam.

## Discussion

In riverine ecosystems, both planktonic and benthic algae are the important components of primary producers. Previous studies mainly focused on species composition and richness of planktonic algae, and their responses to pollutions, disturbance, as well as physic-chemical factors in large rivers [18–22]. In this study, we implemented the synchronous sampling of water and sediment in the Yangtze River, which enables to reveal integrative structure of planktonic and benthic diatoms, and environmental drivers for their biogeographic patterns.

Taxonomic compositions of riverine diatoms in the Yangtze River are markedly different from those in lakes [41] and oceans [13] because of the distinct differences in salinity and hydraulic conditions. For example, *Actinocyclus spp.*, *Aulacoseira spp.*, *Cyclotella spp.*, *Fragilaria spp.*, and *Synedra spp.* predominated in diatom communities in Lake Kasumigaura, Japan [41]. Moreover, *Chaetocero*s, followed by *Fragilariopsis*, *Thalassiosira*, and *Corethron* were reported to be the most abundant genera in the global oceans [13]. In marine ecosystems, diatoms were abundant in nutrient-rich coastal zones particularly at high latitudes. In the riverine ecosystem, diatoms of glacier origin from the Qinghai-Tibetan plateau were significantly different to those in the main body of the Yangtze River (Fig. 2, Additional file 1: Figure S4 and Figure S9). In order of abundance, the most common riverine diatoms in water and sediment of the Yangtze River were *Cyclotella*, *Navicula*, *Pinnularia*, *Stephanodiscus*, and *Cymbella* genera.

Synchronous water and sediment sampling along the Yangtze River makes it possible to compare the biogeography of planktonic and benthic diatoms. On the one hand, although the diversity of planktonic and benthic diatoms was very similar in non-plateau area (Additional file 1: Figure S3), their community composition was remarkably different. Planktonic diatoms were dominated by *Cyclotella* (average relative abundance: 17.66%), *Stephanodiscus* (12.81%), *Pinnularia* (7.53%), *Paralia* (7.34%), and *Skeletonema* (4.90%), whereas benthic diatoms were dominated by *Navicula* (13.12%), *Cyclotella* (10.33%), *Pinnularia* (10.12%), *Surirella* (7.10%), and *Stephanodiscus* (6.53%). On the other hand, seasonal differences were more evident in planktonic diatoms than benthic diatoms (Additional file 1: Figure S4-S6). One-way ANOVA analysis confirmed that water temperature of the Yangtze River was significantly different between spring and autumn (*P <* 0.01), with the water temperature (average 21 °C) in autumn being more conducive than that (average 11 °C) in spring for planktonic diatom growth [42]. Moreover, seasonal fluctuations in water discharge appear to affect the community structure of planktonic diatoms, owing to the introduction of diatom species from the upstream freshwater source and to different hydrologic processes in spring and autumn [43]. Meanwhile, the weak seasonal difference of diatom communities in sediment may be ascribed to the majority of benthic diatoms (Additional file 1: Figure S6) that weakly respond to seasonal changes and reached a state of relative equilibrium through long-term sediment erosion and deposition processes [30].

Interaction between planktonic and benthic diatoms has been one of the major concerns for large river ecosystems. In the Yangtze River, such interactions have several consequences. First, the community composition of planktonic community was significantly correlated with that of benthic diatoms in paired water and sediment samples (in spring: Spearman *r* = 0.3556, *P* = 0.001; in autumn: Spearman *r* = 0.1902, *P* = 0.006). Typical benthic diatoms (e.g., *Nitzschia* and *Navicula*) were found in high abundance in the water column. The local interactions could cause benthic and planktonic habitats to become coupled through migration of algal cells, meaning that phytoplankton can be derived from benthic diatoms, and sinking planktonic algae can become benthic algae [44]. Second, the richness of planktonic diatoms appeared to fluctuate with PAR, whereas the richness of benthic diatoms tended to change with temperature. Nutrient level (TN:TP) had different effects on the diversity and variation of planktonic and benthic diatoms. These phenomena further explain the local interactions in terms of light, temperature, and nutrient competition [45]. Third, given the “River continuum concept” [46] and “Continuous discontinuity concept” [47], the dominance of benthic or planktonic algae changes with the natural riverine gradient (e.g., channel slope), and interactions are invariably interrupted by anthropogenic disturbances such as dam construction and pollutants discharge. Damming has been shown to cause various changes in hydraulic conditions [48–50], such as sediment erosion, deepening river channel, coarse riverbed, and high-suspended particles, which makes the downstream sediments become a habitat with limited light, difficult to adhere, and unsuitable for benthic diatoms to thrive (Additional file 1: Figure S15). Furthermore, it not only weakens the competitive interactions for light and nutrients between benthic and planktonic diatoms, but also reduces their migration rate. Namely, as river channel deepens, the number of planktonic diatoms from detached benthic algae and benthic diatoms from sinking planktonic algae will reduce, resulting a dominant position of primary production by planktonic algae [45]. It can be further confirmed, that is, contrary to the planktonic diatoms, the average richness of benthic diatoms immediately downstream of Three Gorges Dam is significantly lower than that of upstream reaches. Other factors affecting interactions, such as river velocity, turbulent diffusion, algal sinking, and grazing remain to be further investigated [51].

The Yangtze River flows through a variety of landforms such as plateau, mountain, foothill, basin, foothill-mountain, and plain regions along the river continuum [30]. Planktonic and benthic diatoms demonstrate spatial dissimilarities in different types of landforms corresponding to varying soil property, temperature, altitude, light, and nutrients. For example, the Qinghai-Tibet Plateau is characterized with lower temperature, higher altitude, and the greatest PAR, while the basin (mostly located in Sichuan province) receives the lowest light resource. Further going down to the Middle-Lower Yangtze Plain, there exists a general decrease of altitude and increase of temperature and nutrient levels (nitrogen and phosphorus). The differentially abundant diatoms in both water and sediments in the six landform types could also be convinced by previous studies on special diatoms in different landforms such as mountain [52] and plateau [53]. Similar findings have also been reported recently on bacterial communities. For example, Liu et al. [30] confirmed that landform type is of significance to bacterial structures in the Yangtze River. Moreover, Chen et al. [54] indicated that anammox bacterial abundance and alpha diversity are spatially subject to landform variations.

It remains a practical challenge to examine the effects of environmental selection and spatial dispersal on determining algae communities in river ecosystems. In the Yangtze River, environmental selection appears to explain at least three times more of the community variance (either in planktonic or benthic diatoms) than spatial dispersal process (Additional file 1: Figure S12), which is in line with previous findings on planktonic [26] and benthic diatoms [28] in streams and rivers. Keck et al. reported the importance of both environment and dispersal-related processes in controlling planktonic diatom community structure in stream and rivers, Sweden [26]. Soininen et al. concluded that local environmental factors were even more important than spatial factors in explaining benthic diatom distributions in boreal streams, Finland [28]. Overall, environmental selection is the dominant driving force on the biogeographical pattern of planktonic and benthic diatom communities in the Yangtze River.

The environmental-based selection process driving the biogeographic pattern of diatoms is influenced by PAR, temperature, channel slope, and nutrient level. The community structure of benthic diatoms is a typical consequence driven by environmental selection in the Yangtze River. First, it has been reported that sufficient PAR drives the growth and production of diatoms [55], but excess PAR can affect various cellular processes and reduce the growth or viability of diatoms [56]. In the present study, specific diatoms were identified as preferring to different levels of light intensity (Fig. 3), indicating that diatoms possess diverse light-regulatory mechanisms and adaptive responses [57]. Although both light and temperature are essential for diatom growth, planktonic and benthic diatoms exhibit different preferences for PAR and water temperature. In the surface oceans, planktonic diatoms have been shown to be replaced by small phytoplankton, causing decreased primary production and carbon export, due to global warming [58]. In the Yangtze River, we observed that the richness of benthic diatoms tended to rise with increasing temperature (Fig. 3c). Moreover, the composition of benthic diatoms appeared to be strongly influenced by temperature (Additional file 1: Table S3), suggesting that benthic diatoms are more sensitive to temperature changes than planktonic diatoms in river ecosystem.

Second, as a primary driver of stream power that shapes the spatial distribution of diatoms, the channel slope not only affects competitive and succession processes among species but also alters nutritional utilization strategies and hence the production and growth of diatoms [59], leading to diatoms preferring to different flow conditions [60]. In benthic diatoms, over 40% abundance was taken by motile-guild species capable of moving fast and choosing the best microhabitat under certain circumstances (Fig. 4c). In other words, the motile guild can respond quickly to environmental changes, and benthic diatoms dominated by motile guild are more appropriate to reflect the influence of environmental heterogeneity along the river.

Third, the weak relationship between annual-averaged TN:TP and alpha-diversity of planktonic diatoms along the Yangtze River might be resulted from the selection of simpler indicator for nutrient level in a relatively oligotrophic river (Additional file 1: Figure S14). In fact, other environmental factors such as hydrodynamic condition, light, temperature, and biological predation may also play considerable roles over the full year. Moreover, species-specific responses to nitrogen and phosphorus in the production and growth of diatoms would be more complex. For example, *Nitzschia palea* [61] is phosphorus limited, but *Chaetoceros calcitrans* [62] is efficient in nitrogen assimilation, regardless the unclear mechanism on diatom utilization of nitrogen and phosphorus in different forms [63, 64].

Finally, damming in rivers is a typical anthropogenic perturbation which could profoundly modify material fluxes and biogeochemical cycles of downstream [48, 49]. Kunz et al [48] reported that sediment, carbon, nitrogen, and phosphorus were trapped by the reservoir immediately upstream of the Itezhi-Tezhi Dam, increasing the N:P ratio downstream of the dam. High flow downstream of dams has caused severe erosion of the riverbed and led to coarsening of bed materials [49]. Changes in water level also affect the light intensity and temperature to benthic diatoms. In short, a large dam disrupts the hydraulic gradient, nutrient conditions, light availability, and temperature in rivers, resulting in local changes to the environment in which benthic diatoms thrive (Additional file 1: Figure S15).

## Conclusions

This study provided the first molecular biogeographic pattern of both planktonic and benthic diatoms over a continuum of 6030 km in the Yangtze River. Temporally, planktonic diatom communities demonstrated significant seasonal difference in the mainstream, and spatially dominant diatoms in water and sediment varied with landforms, such as the plateau, mountain, foothill, basin, foothill-mountain, and plain, from the river source to the estuary of the Yangtze. Comparing with the spatial dispersal process, the environmental selection process was the major driver for diatom biogeography, which could be further interpreted in terms of photosynthetically active radiation, hydraulic slope, nutrients, and human activities (the Three Gorges Dam and the Xiluodu Dam). Our study revealed that benthic diatoms represented by motile species in ecological guilds are typical consequences driven by environmental selection in a lotic-oligotrophic river, which highlights the particular importance of benthic diatoms in understanding biogeochemical cycles in world’s large river ecosystems.

## Methods

### Sample collection

The Yangtze River is the longest river situated wholly in Asia and the third longest in the world, with a drainage basin of 1.8 million km^2^. The river is over 6300 km long, has its source in the Qinghai-Tibet Plateau, and flows eastwards into the East China Sea near Shanghai. Over its length, the Yangtze River experiences great changes in landform type and hydrological regime and supports more than 588 million people [65].

In March (spring) and October (autumn) of 2014, water and sediment samples were synchronously (i.e., within 1 week) collected for planktonic and benthic diatom identification at 50 national monitoring stations (the non-plateau area) along the mainstream and six major tributaries of the Yangtze River. Finally, 84 water samples (i.e., 38 water-spring samples and 46 water-autumn samples) and 171 sediment samples (i.e., 87 sediment-spring samples and 84 sediment-autumn samples) were obtained for studying the seasonal and spatial distribution of diatom communities in the non-plateau area. To investigate the difference of diatoms in the plateau and the non-plateau areas, we implemented an additional synchronous monitoring along the source river of the Yangtze located at the Qinghai-Tibet Plateau in July 2017 (the ideal month for sampling in the plateau), collected 12 water and 12 sediment samples respectively at 12 sites. Although the most ideal sampling should be the synchronous monitoring in both plateau and non-plateau areas in the same year, the above remedial sampling is helpful considering insignificant inter-annual variations of water quality, riverine habitat, and aquatic organism in the plateau in recent years [66, 67]. Except for a very few samples missed due to restrictions of steep terrain and rapid flow as described in a previous study [30], up to four parallel samples were collected in most cases. At each sampling site, 10 L of well-mixed water was collected and then filtered onto 0.22 μm polycarbonate membranes (Millipore, USA) within 24 h. Filter membranes and sediment samples were stored in the laboratory at – 80 °C until further analysis took place.

### Measurement of environmental variables

Environmental parameters including water temperature, chemical oxygen demand (COD), suspended solids (SS), dissolved oxygen (DO), pH, ammonium nitrogen (NH_4_-N), nitrate nitrogen (NO_3_-N), total nitrogen (TN), total phosphorus (TP), and dissolved organic carbon (DOC) were measured for water samples according to Environmental Quality Standards for Surface Water (GB3838-2002) recommended by the Ministry of Ecology and Environment of China. For sediment samples, pH, total organic carbon (TOC), NH_4_-N, NO_3_-N, TN, and TP contents were measured as described by Zhu et al. [68]. For each sampling sites, longitude, latitude, and altitude were recorded by a handheld GPS (Magellan, USA).

Dissolved carbon dioxide (*p*CO_2_) in water was measured using a headspace equilibration technique [69]. In short, 75 mL of river water was collected in a 100 mL polypropylene syringe. Air bubbles drawn in with the sample were eliminated by tapping and draining water while the syringe is pointing upwards. Dissolved CO_2_ was extracted by transferring 25 ml of ultra-high purity nitrogen at the field sites and then equilibrated with the headspace in the sample syringe by vigorous shake for 5 min. After equilibration, the headspace gas was immediately transferred to a pre-vacuum glass storage vial equipped with chlorobutyl septa. Finally, the CO_2_ partial pressure was measured using a gas chromatograph equipped with a thermal conductivity detector.

The information on channel slope in the Yangtze was sourced from Chen et al. [70]. Nutrients condition was represented by the atomic ratio of nitrogen to phosphorus. For water samples, we utilized dissolved total nitrogen (TN) and total phosphorus (TP) to calculate annual-averaged TN:TP for 2005 to 2014 and monitored TN:TP observed at 50 stations for 2014. For sediment samples, we utilized TN and TP to calculate monitored TN:TP observed at 50 stations for 2014.

### DNA extraction, PCR amplification, and sequencing

DNA was extracted in triplicate using the FastDNA® SPIN Kit for Soil (MP Biomedicals, USA) following the manufacturer’s instructions. The triplicate DNA extracts were mixed together for later PCR amplification. Amplification of the V4 region of the 18S rDNA was performed by polymerase chain reaction (PCR) using barcoded primers DIV4for (5′-GCGGTAATTCCAGCTCCAATAG-3′) and DIV4rev3 (5′-CTCTGACAATGGAATACGAATA-3′) [12], where barcode is an eight-base sequence unique to each sample. Amplification was conducted under the following conditions: initial denaturation at 94 °C for 2 min, then 32 cycles of denaturation at 94 °C for 45 s, annealing at 50 °C for 45 s, elongation at 72 °C for 60 s, and final extension at 72 °C for 10 min, 10 °C until halted by user. PCR mixtures (20 μL volume) were prepared in triplicate contained 2 μL of 10 × buffer, 2 μL of 2.5 mM dNTPs, 0.8 μL of each primer (5 μM), 0.2 μL of rTaq polymerase, 0.2 μL of BSA, and 1 μL of 10 ng DNA sample. Amplicons were purified using the AxyPrep DNA Gel Extraction Kit (Axygen Bioscience, Union City, CA, USA) according to the manufacturer’s instructions and quantified using QuantiFluorTM-ST (Promega, USA). Adaptor was ligated onto the amplicons for the library construction. Afterwards, sample libraries were pooled in equimolar amounts and sequenced on Illumina MiSeq 2 × 250 PE platform (Majorbio Company, Shanghai, China).

Three negative control samples were used to monitor any contamination during the molecular workflow, negative filtration, DNA extraction, and PCR controls; however, no quantifiable DNA was detected for further analysis.

### Bioinformatics analysis

Sequences of diatom 18S rDNA were quality-filtered using QIIME [71] as follows: (i) minimum sequence length of 300 bp, and minimum threshold quality score of Q20; (ii) maximum mismatches of 2 for matching the primer; any reads with ambiguous bases were removed; and (iii) merged pair-ended sequences that overlapped longer than 10 bp into a single sequence. UCHIME was used to remove chimeric sequences and UPARSE was used to cluster operational taxonomic units (OTUs) with 97% similarity cutoff [72].

We built a reference database of 18S rRNA reads composed of 4573 unique diatom sequences. First, we extracted all diatom sequences of 18S rRNA reads from GenBank (http://www.ncbi.nlm.nih.gov/). Second, short reads (less than 100 nucleotides) were refused access to the reference database, and redundant reads were eliminated by cd-hit to increase the taxonomy identification accuracy. Third, sequence alignment was performed by Mafft (ver 7.310) [73], then the sequences were analyzed to construct an approximately-maximum-likelihood phylogenetic tree using FastTree (ver 2.1.10) [74], and any incorrect reads discarded. Finally, a total of 4573 unique sequences were retained in our reference database.

To identify taxonomically OTUs obtained in this study against known diatom species, the BLASTN [75] program was applied to align clean 18S rRNA reads to the corrected diatom database. Those OTUs with the best BLAST hit scores, not only an *e* value ≤ 10^−5^ but also identity ≥ 80% with respect to the reference sequence were firstly selected. Then, the selected OTUs were checked by means of the phylogenetic tree, and only OTUs with correct taxonomical assignment were retained for further analysis. Clean reads were further assigned to known diatom species based on our reference database.

To estimate the community structure for each site, the Mothur program [76] was used to normalize all data sets with respect to the least-well-represented data set (11049 sequences). Alpha diversity indices (chao1, Shannon, and Goods coverage) were calculated using QIIME.

### Statistical analysis

Diatom species that characterize each sample group were identified with indicator species analysis using labdsv and indval packages in R software [77]. Indicator values were calculated based on the relative frequency and relative average abundance of a given species in six types of environmental samples. Species with indicator value ≥ 0.3 and *p* value ≤ 0.01 were defined as indicator species at class, order, family, and genus levels. Nonmetric multidimensional scaling (NMDS) was performed to visualize the dissimilarity of different samples based on Bray-Curtis similarity matrices. Analysis of similarity (ANOSIM) was conducted to test the significance of differences among a priori sampling groups based on environmental parameters. NMDS and ANOSIM statistics were carried out using the vegan package in R. The linear discriminant analysis effect size (LEfSe) [36] was used with Kruskal-Wallis and Wilcoxon tests to discover high-dimensional biomarker and explain taxa difference at different environment conditions of PAR or channel slope. The LEfSe biomarker detection was performed in QIIME [71] using the logarithmic LDA threshold > 4 and the statistical parameters of *P* < 0.05. One-way analysis of variance (one-way ANOVA) was carried out to test significance of group differences using the vegan package in R.

Distance-decay patterns of diatom community similarity were described by considering geographical distance and environmental distance from the site location to river mouth among sample sites. Mantel tests were used to examine the Spearman’s rank correlation between geographical and environmental distance and diatom community similarity using Bray-Curtis distance matrices with 999 permutations in R. The geographical distance of each sampling site was calculated using ArcGIS V10.3 software. The environmental distance matrix (normalized Euclidean distance) was generated with a normalized combination of environmental variables such as water temperature, COD, SS, DO, pH, NH_4_-N, NO_3_-N, TN, TP, and DOC for water samples as well as TOC, pH, NH_4_-N, NO_3_-N, TN, and TP for sediment samples. The rate of distance-decay of diatom communities was calculated as the slope of ordinary least-squares regression line fitted to the relationship between geographic distance and community similarity. Partial Mantel tests were conducted to assess the pure effects of geographical distance (controlling for environmental distance) and environmental distance (controlling for geographic distance) on diatom community similarity with 9999 permutations.

A set of spatial variables was generated through the use of principal coordinates of neighbor matrices (PCNM) analysis based on the longitude and latitude coordinates of each sampling site [78]. The function “envfit” was run with 999 permutations to select significant environmental variables (*P* < 0.05). Significance testing was then assessed using the “permutest” function based on 999 permutations in R, while canonical correspondence analysis (CCA) was performed to determine the effects of selected environmental and spatial variables on diatom communities (Additional file 1: Table S3). Partial canonical correspondence analysis (pCCA) was performed to decompose the total variation in diatom community into a pure environmental component, a pure spatial component, a spatially structured environmental component, and residual variation.

### Ecological guild classification

Based on their ecological characteristics, diatom species are classified into four ecological guilds (low profile, high profile, motile, and planktic guilds) [31–33], which are expected to respond in different ways to nutrients’ conditions and physical disturbances. A low-profile guild is defined as having high reproduction rate, low nutrient and light availability, and slow-moving diatoms. A high-profile guild possesses characteristics of high resource availability and low disturbance. A motile ecological guild has the ability to move fast and choose the best microhabitat in a given circumstance. A planktic guild adapts to lentic environments and resists sedimentation. We extended these guilds by adding supplemented classifications used in other studies [79, 80].

### Photosynthetically active radiation (PAR) divisions

Solar radiation with wavelengths (400–700 nm), called photosynthetically active radiation (PAR), is able to be utilized by plants and algae through photosynthesis to convert light energy into biomass [34]. Monteith reported the linear correlation between net primary production (NPP) and PAR absorbed by green foliage [81]. Zhu et al. [35] also suggested that the spatial distribution of annual-averaged PAR is complex and inhomogeneous across China, using calculated PAR spatial data for the period 1961–2007 provided by China Meteorological Administration.

Zhu et al. [35] calculated and spatialized PAR using data simulation method [82] based on three climatic datasets, i.e., daily sunshine duration data at 740 weather stations across China for 1961–2007 and global radiation data at 122 radiation stations across China for 1961–2000 from China Meteorological Administration, and PAR observatory data at 36 field stations across China for 2004–2007 from Chinese Ecosystem Research Network. Then, PAR along the Yangtze River could be further derived. Thus, we define four zones of PAR intensity in different regions across the Yangtze River basin as follows:

I. Very high, PAR > 32 mol m^−2^ d^−1^;
II. High, 26 < PAR ≤ 32 mol m^−2^ d^−1^;
III. Medium, 23 < PAR ≤ 26 mol m^−2^ d^−1^;
IV. Low, PAR ≤ 23 mol m^−2^ d^−1^.

## Supplementary information

**Additional file 1:** **Figure S1.** Rarefaction curves of diatom richness for 279 samples. **Figure S2.** Phylogenetic distribution of reference sequence and abundant OTUs. **Figure S3.** Alpha diversity index per diatom community for each sample type. **Figure S4.** Nonmetric multidimensional scaling diagram of Bray-Curtis dissimilarities between diatom communities for all samples. **Figure S5.** ANOSIM statistics concerning differences in diatom communities within and between sample types. **Figure S6.** Diatom genera exhibiting significant seasonal differences in water (**a**) and sediment (**b**) samples. **Figure S7.** Spearman relationships between diatoms and dissolved carbon dioxide. **Figure S8.** Biogeographical distribution of diatoms at class level throughout the mainstream of the Yangtze for: (**a**) water-spring, (**b**) water-autumn, (**c**) sediment-spring, and (**d**) sediment-autumn samples. **Figure S9.** Proportion of indicator diatoms in each sample type obtained using indicator taxa analysis at Class (**a**), Order (**b**), Family (**c**), and Genus (**d**) levels. **Figure S10.** Biogeographical distribution of ecological guilds throughout the mainstream of the Yangtze River. **Figure S11.** Distance-decay relationships between community similarity and geographic distance for water (**a**) and sediment (**b**) samples. **Figure S12.** Variation in community composition explained by environmental, spatial, and spatially structured environmental components. **Figure S13.** LEfSe cladogram of planktonic (**a**) and benthic (**b**) diatom communities for the three channel slope regions. **Figure S14.** Relationships between Shannon diversity and TN:TP for water-spring (**a**), water-autumn (**b**), sediment-spring (**c**), and sediment-autumn (**d**) samples. Distance relationship of TN:TP for sampling sites along the mainstream (**e**). **Figure S15.** Significant differences in abundance of benthic diatoms upstream and downstream of Xiluodu Dam (**a**) and Three Gorges Dam (**b**). **Table S1.** Numbers of Indicator species and Top Indicator species across sample sites. **Table S2.** Partial Mantel test for Spearman correlations between community similarity and geographic and environmental distances. **Table S3.** Effects of selected factors on diatom community using canonical correspondence analysis.

## Data Availability

Complete datasets supporting the findings of this article are in the NCBI Sequence Read Archive (SRA) database (Accession Number: SRP153344).

## Abbreviations

OTUs: Operational taxonomic units
HTS: High-throughput sequencing
NMDS: Nonmetric multidimensional scaling
ANOSIM: Analysis of similarity
One-way ANOVA: One-way analysis of variance
LefSe: Linear discriminant analysis effect size
PAR: Photosynthetically active radiation
TN:TP: Ratio of total nitrogen to total phosphate
