# Supplementary material for: Molecular biogeography of planktonic and benthic diatoms in the Yangtze River
Source: Microbiome. 2019 Dec 5;7:153. doi: 10.1186/s40168-019-0771-x (PMC6896584; doi:10.1186/s40168-019-0771-x)
Supplement: Supplementary file 1 — Additional file 1: Figure S1. Rarefaction curves of diatom richness for 279 samples. Figure S2. Phylogenetic distribution of reference sequence and abundant OTUs. Figure S3. Alpha diversity index per diatom community for each sample type. Figure S4. Nonmetric multidimensional scaling diagram of Bray-Curtis dissimilarities between diatom communities for all samples. Figure S5. ANOSIM statistics concerning differences in diatom communities within and between sample types. Figure S6. Diatom genera exhibiting significant seasonal differences in water (a) and sediment (b) samples. Figure S7. Spearman relationships between diatoms and dissolved carbon dioxide. Figure S8. Biogeographical distribution of diatoms at class level throughout the mainstream of the Yangtze for: (a) water-spring, (b) water-autumn, (c) sediment-spring, and (d) sediment-autumn samples. Figure S9. Proportion of indicator diatoms in each sample type obtained using indicator taxa analysis at Class (a), Order (b), Family (c), and Genus (d) levels. Figure S10. Biogeographical distribution of ecological guilds throughout the mainstream of the Yangtze River. Figure S11. Distance-decay relationships between community similarity and geographic distance for water (a) and sediment (b) samples. Figure S12. Variation in community composition explained by environmental, spatial, and spatially structured environmental components. Figure S13. LEfSe cladogram of planktonic (a) and benthic (b) diatom communities for the three channel slope regions. Figure S14. Relationships between Shannon diversity and TN:TP for water-spring (a), water-autumn (b), sediment-spring (c), and sediment-autumn (d) samples. Distance relationship of TN:TP for sampling sites along the mainstream (e). Figure S15. Significant differences in abundance of benthic diatoms upstream and downstream of Xiluodu Dam (a) and Three Gorges Dam (b). Table S1. Numbers of Indicator species and Top Indicator species across sample sites. Table S2. Partial Mant [file 40168_2019_771_MOESM1_ESM.docx]

**Supplementary Material for**

**Molecular Biogeography of Planktonic and Benthic Diatoms in the Yangtze River**

Jiawen Wang^1†^, Qingxiang Liu^1†^, Xianfu Zhao^2^, [Alistair G. L. Borthwick](http://pubs.acs.org/action/doSearch?ContribStored=Borthwick%2C+A+G+L)^3^, Yuxin Liu^1^, Qian Chen^1,4^, Jinren Ni^5*^

**Author Affiliations:**

^1^College of Environmental Sciences and Engineering, Key Laboratory of Water and Sediment Sciences, Ministry of Education, Peking University, Beijing, 100871, China

^2^Institute of Hydroecology, Ministry of Water Resources, Chinese Academy of Sciences, Wuhan 430079, China

^3^Institute of Infrastructure and Environment, School of Engineering, University of Edinburgh, The King’s Buildings, Edinburgh EH9 3JL, UK

^4^State Key Laboratory of Plateau Ecology and Agriculture, Qinghai University, Xining, 810016, China

^5^Beijing Innovation Center for Engineering Science and Advanced Technology, Peking University, Beijing, 100871, P. R. China

^†^Equal contributors

^*^Correspondence: Jinren Ni, College of Environmental Sciences and Engineering, Peking University, Beijing, 100871, China. E-mail: jinrenni@pku.edu.cn


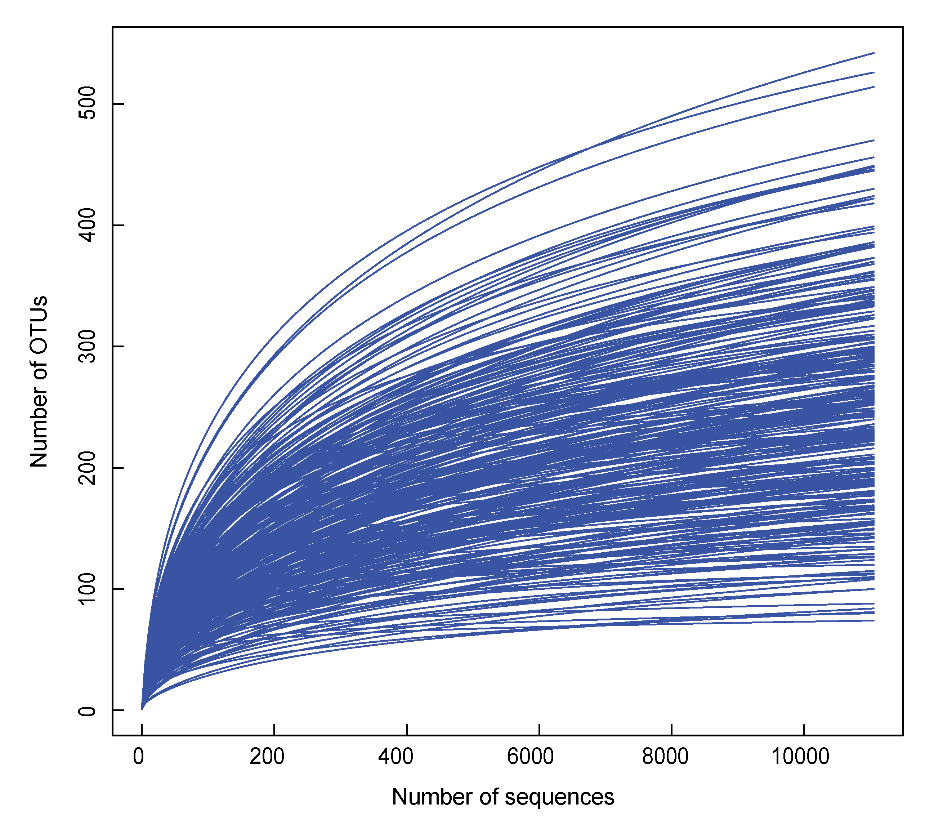


**Figure S1**. Rarefaction curves of diatom richness for 279 samples.


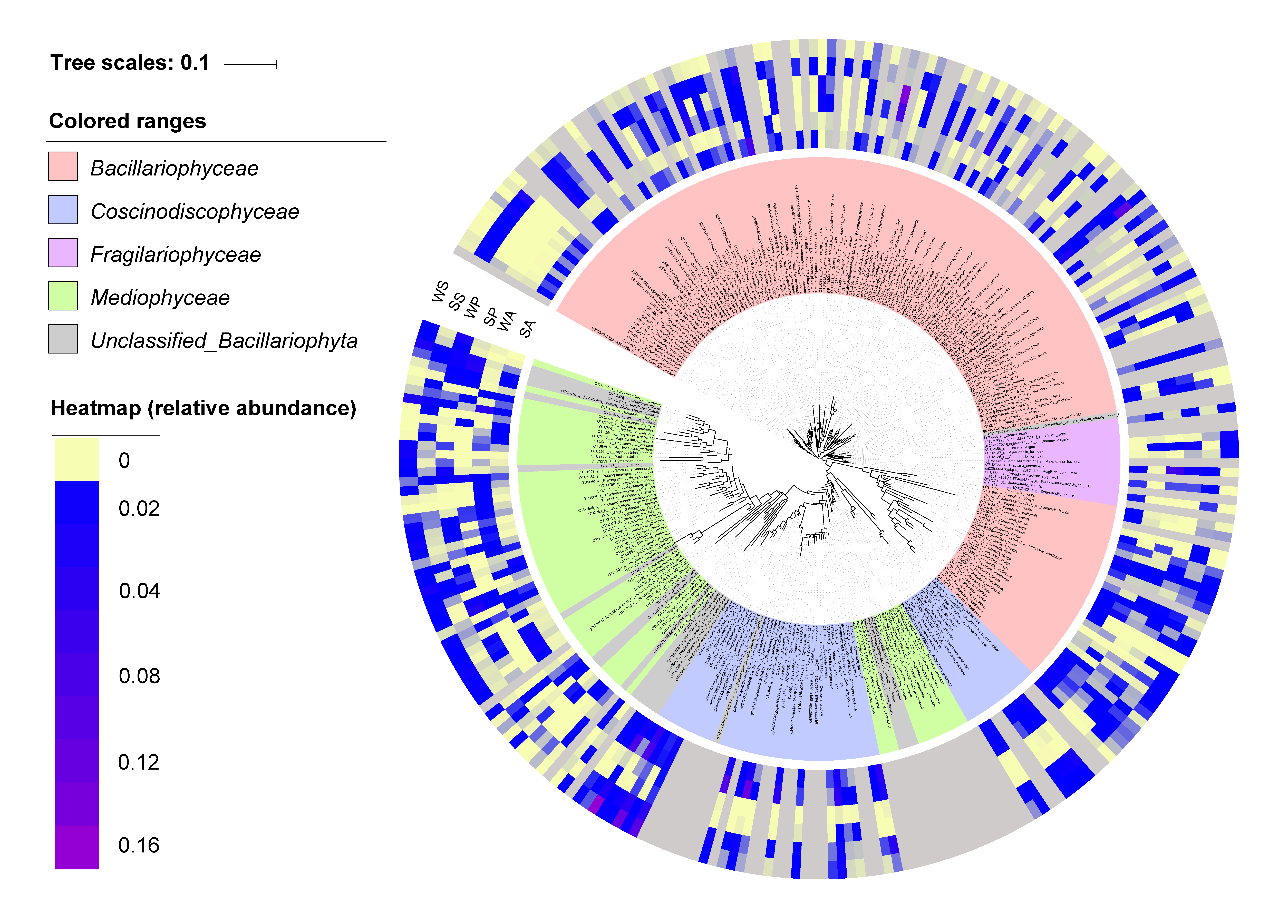


**Figure S2**. Phylogenetic distribution of reference sequence and abundant OTUs. The color range displays class level taxonomy information on the node branch. The outmost heatmap indicates relative abundance of OTUs in six sample types: WS, water-spring; SS, sediment-spring; WA, water-autumn; SA, sediment-autumn; WP, water-plateau; SP, sediment-plateau.


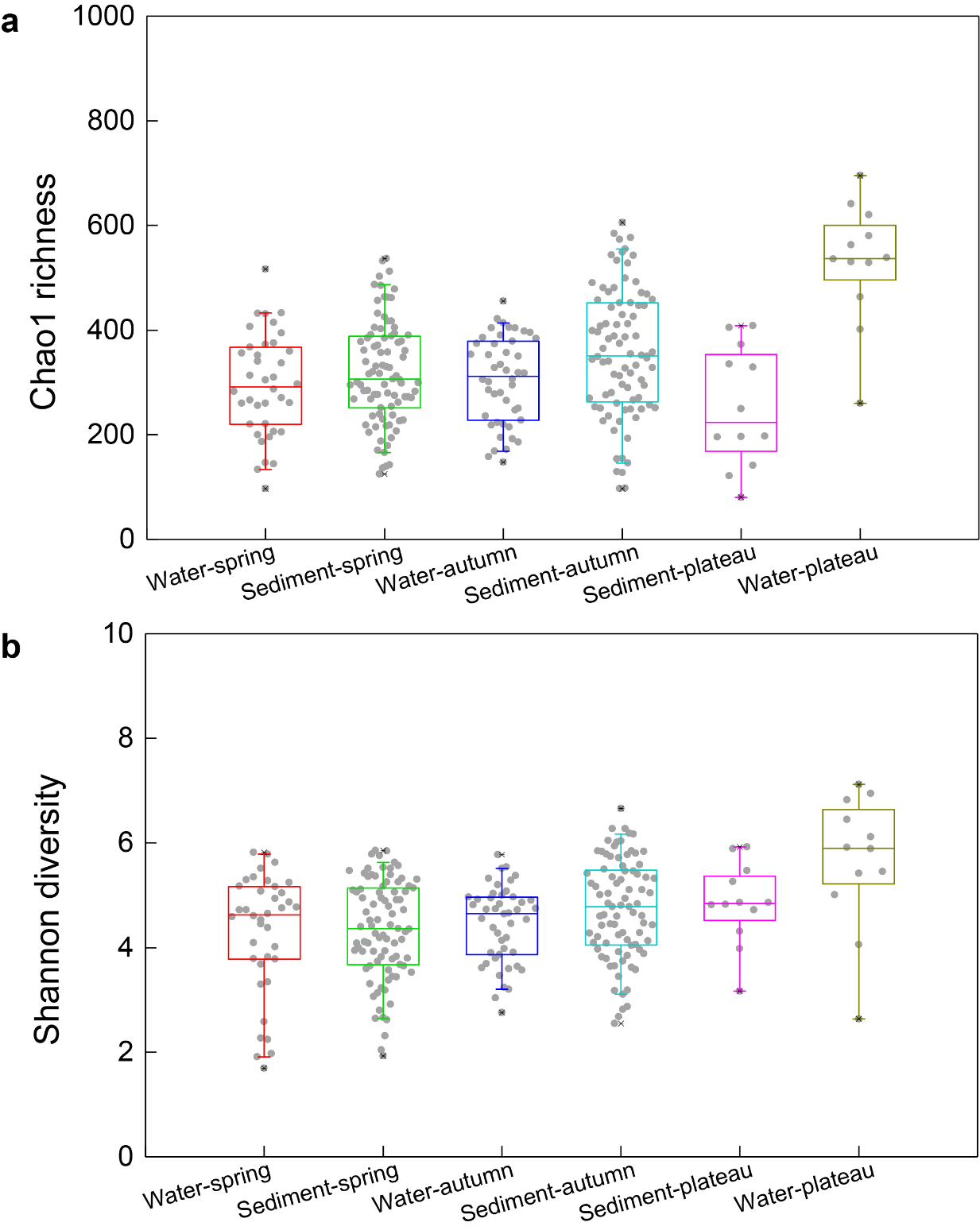


**Figure S3**. Alpha diversity index per diatom community for each sample type.


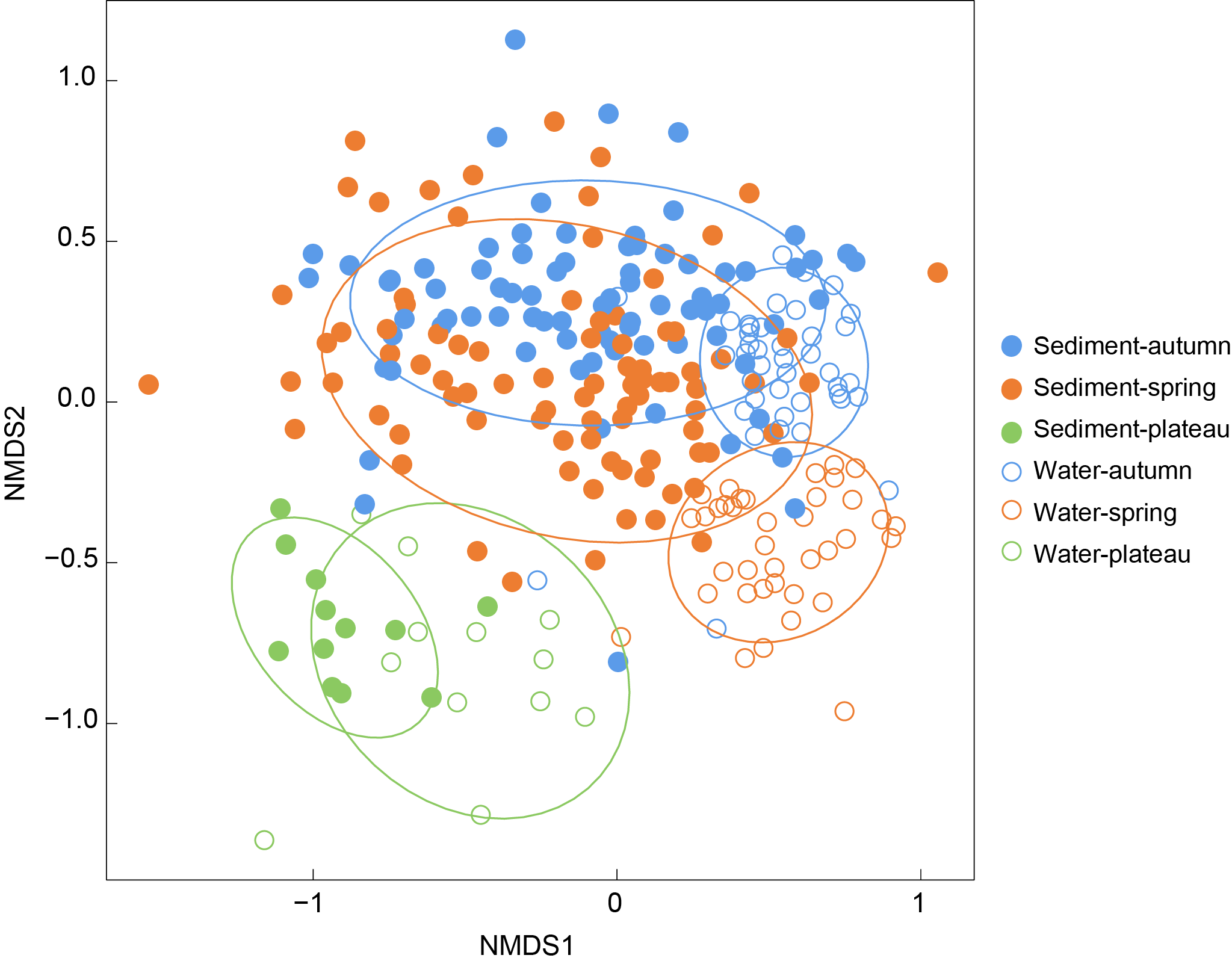


**Figure S4**. Nonmetric multidimensional scaling diagram of Bray-Curtis dissimilarities between diatom communities for all samples.


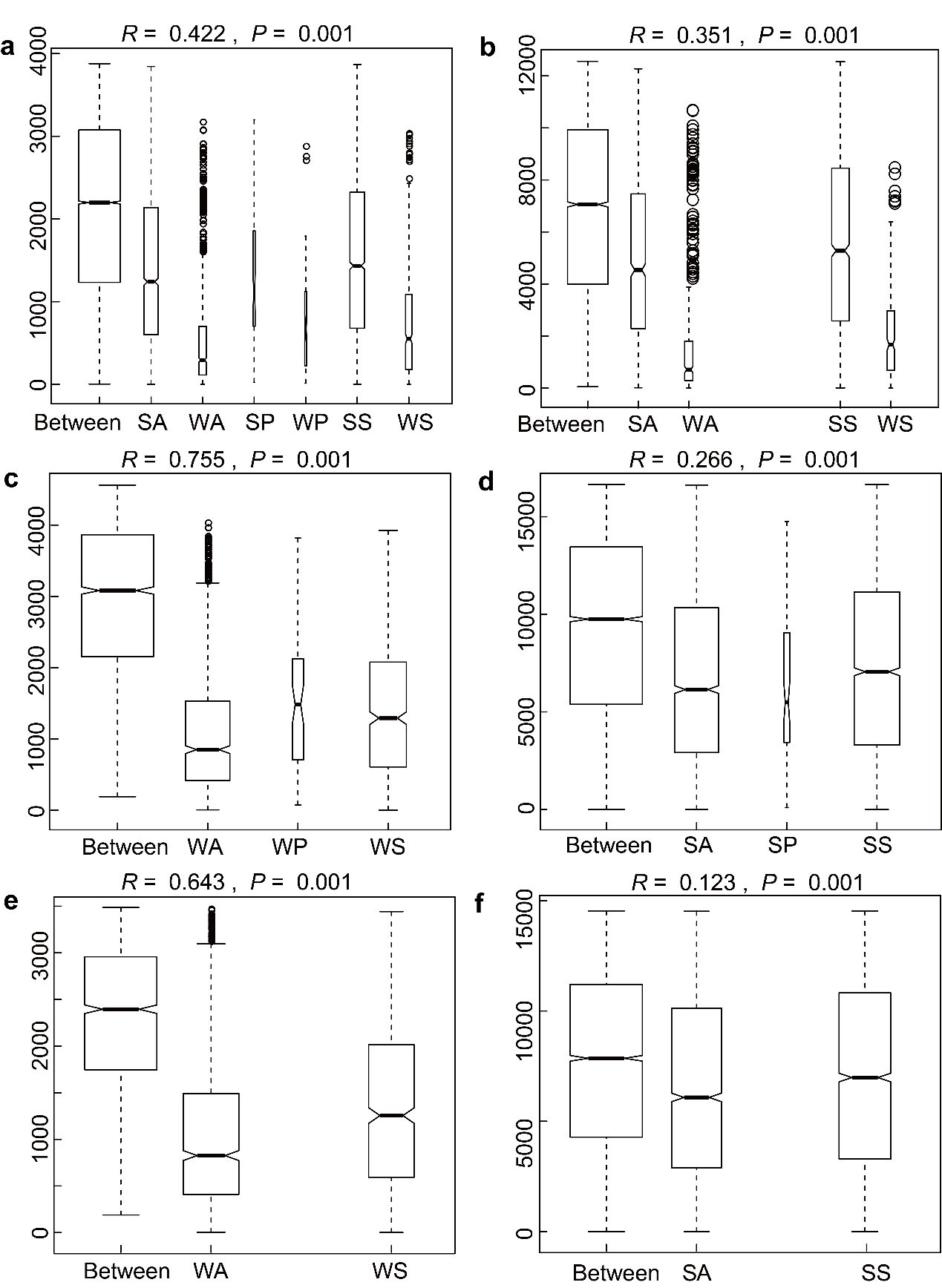


**Figure S5**. ANOSIM statistics concerning differences in diatom communities within and between sample types.


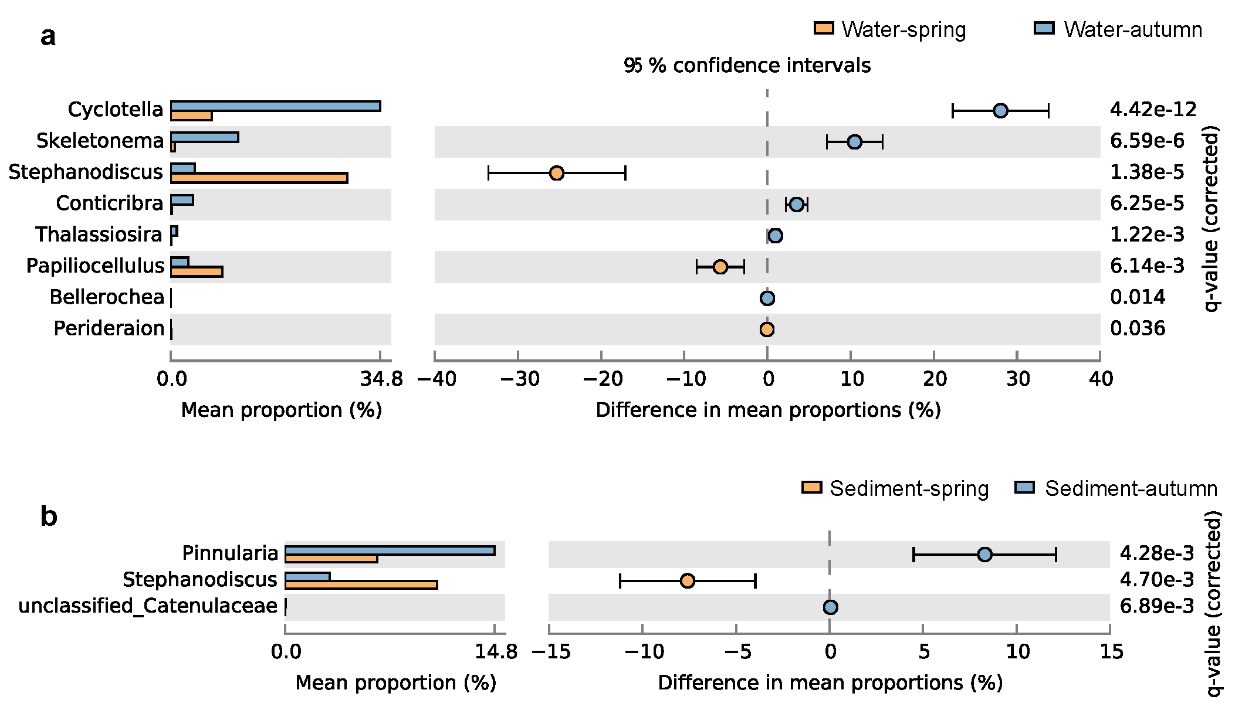


**Figure S6**. Diatom genera exhibiting significant seasonal differences in water (**a**) and sediment (**b**) samples.


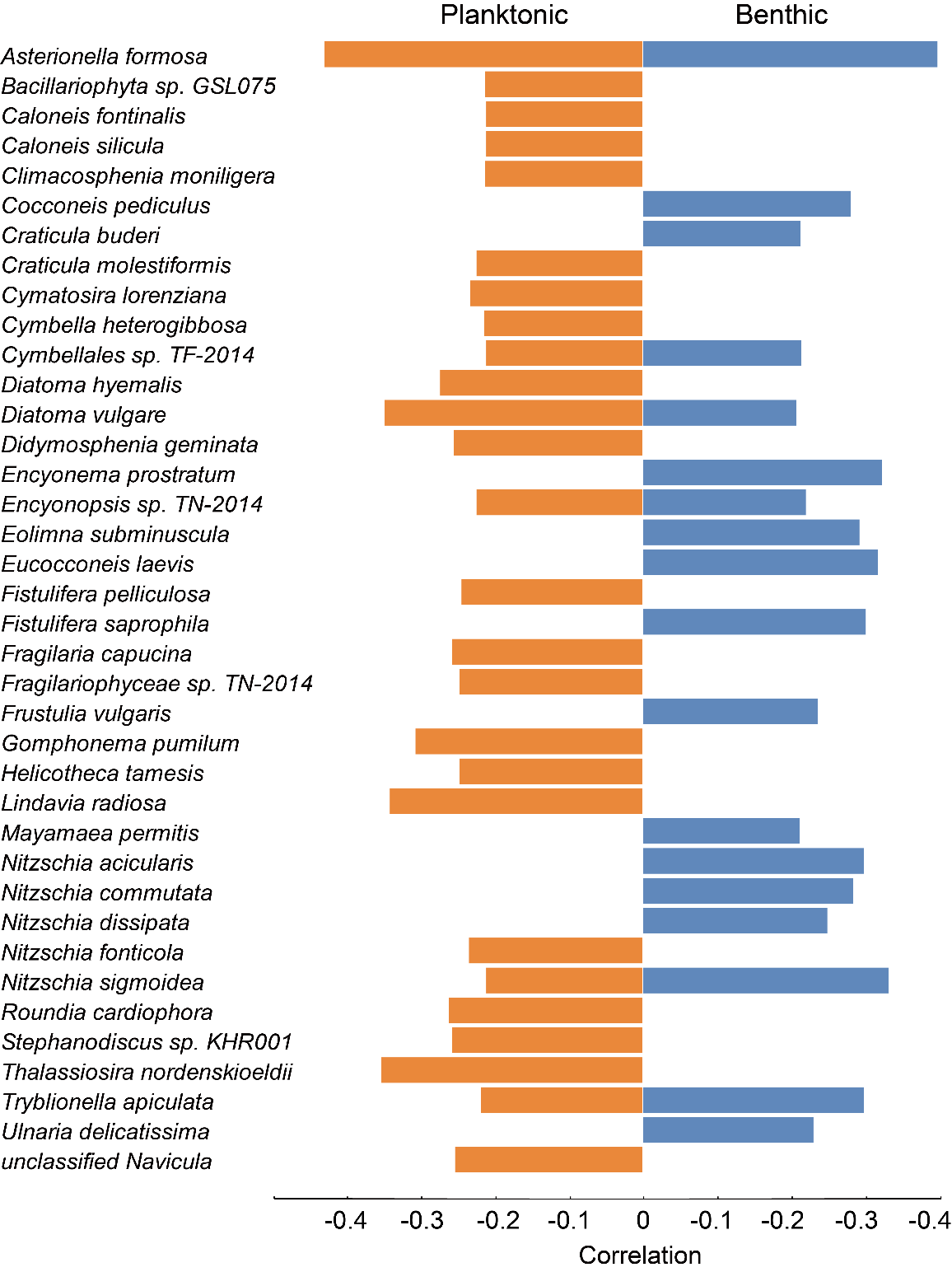


**Figure S7**. Spearman relationships between diatoms and dissolved carbon dioxide.


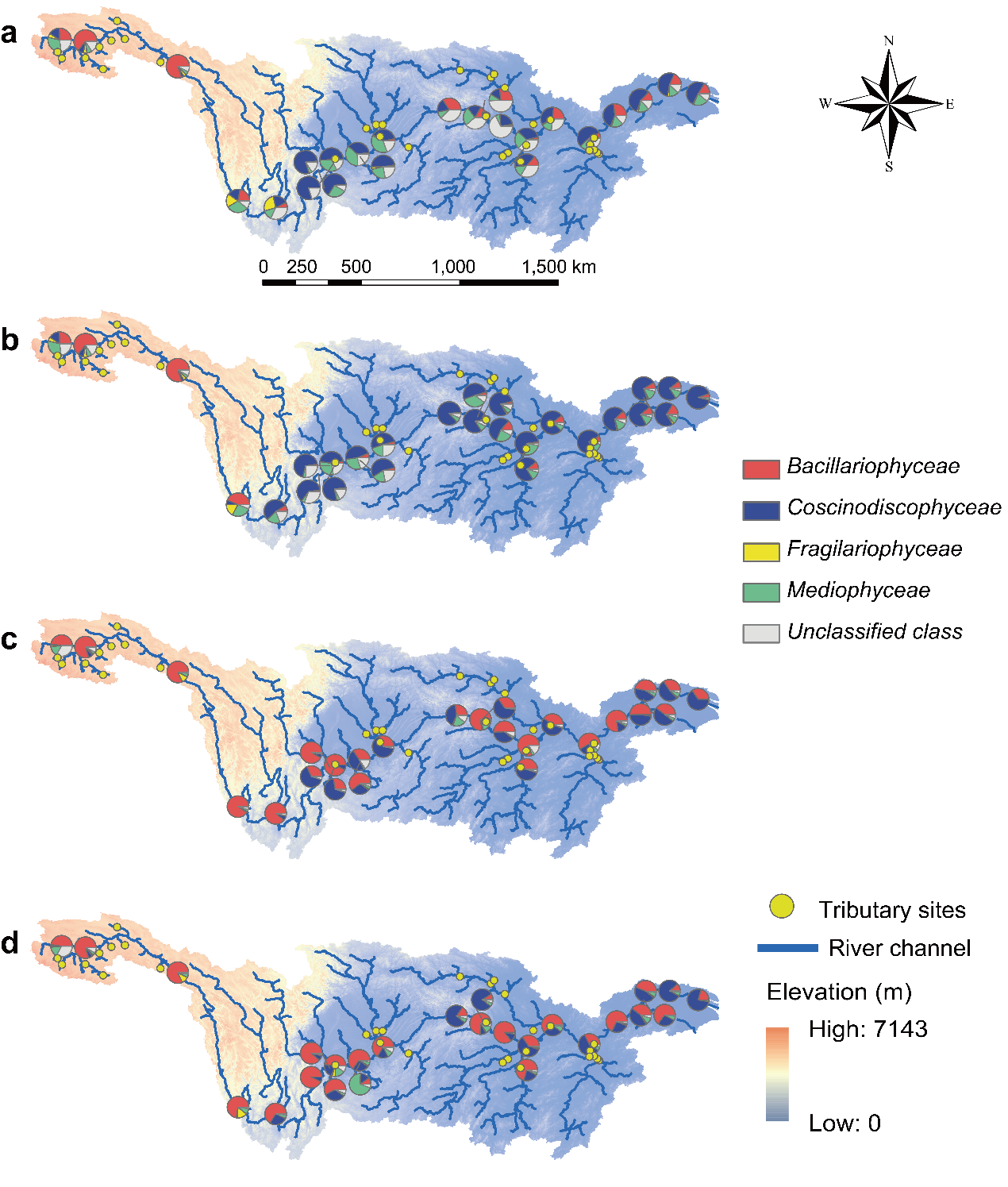


**Figure S8**. Biogeographical distribution of diatoms at class level throughout the mainstream of the Yangtze for: (**a**) water-spring, (**b**) water-autumn, (**c**) sediment-spring, and (**d**) sediment-autumn samples. For comparison, water-plateau samples are displayed in (**a**) and (**b**)**;** sediment-plateau samples are displayed in (**c**) and (**d**).


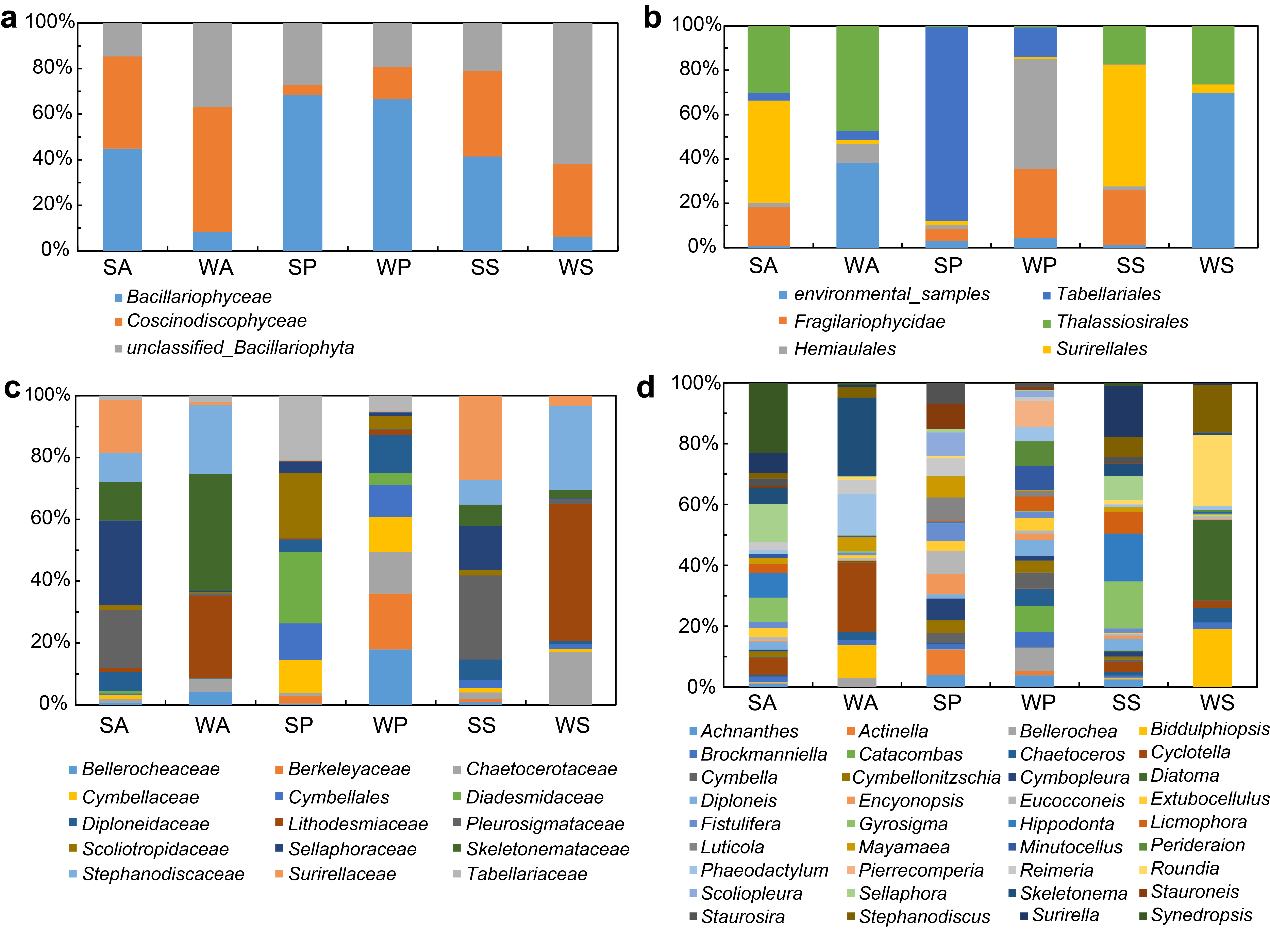


**Figure S9**. Proportion of indicator diatoms in each sample type obtained using indicator taxa analysis at Class (**a**), Order (**b**), Family (**c**), and Genus (**d**) levels.


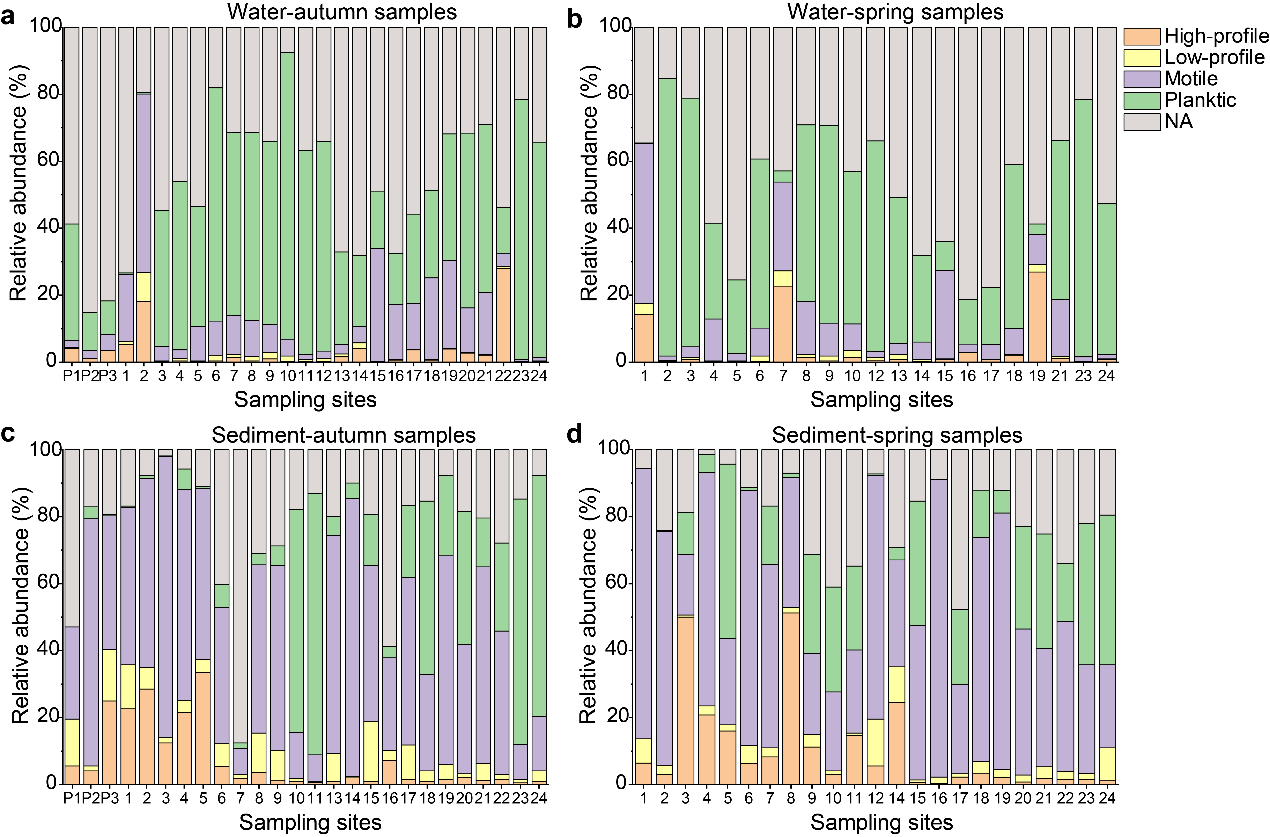


**Figure S10**. Biogeographical distribution of ecological guilds throughout the mainstream of the Yangtze River. For comparison, water-plateau samples (P1-P3) are displayed in (**a**) and (**b**); sediment-plateau samples (P1-P3) are displayed in (**c**) and (**d**).


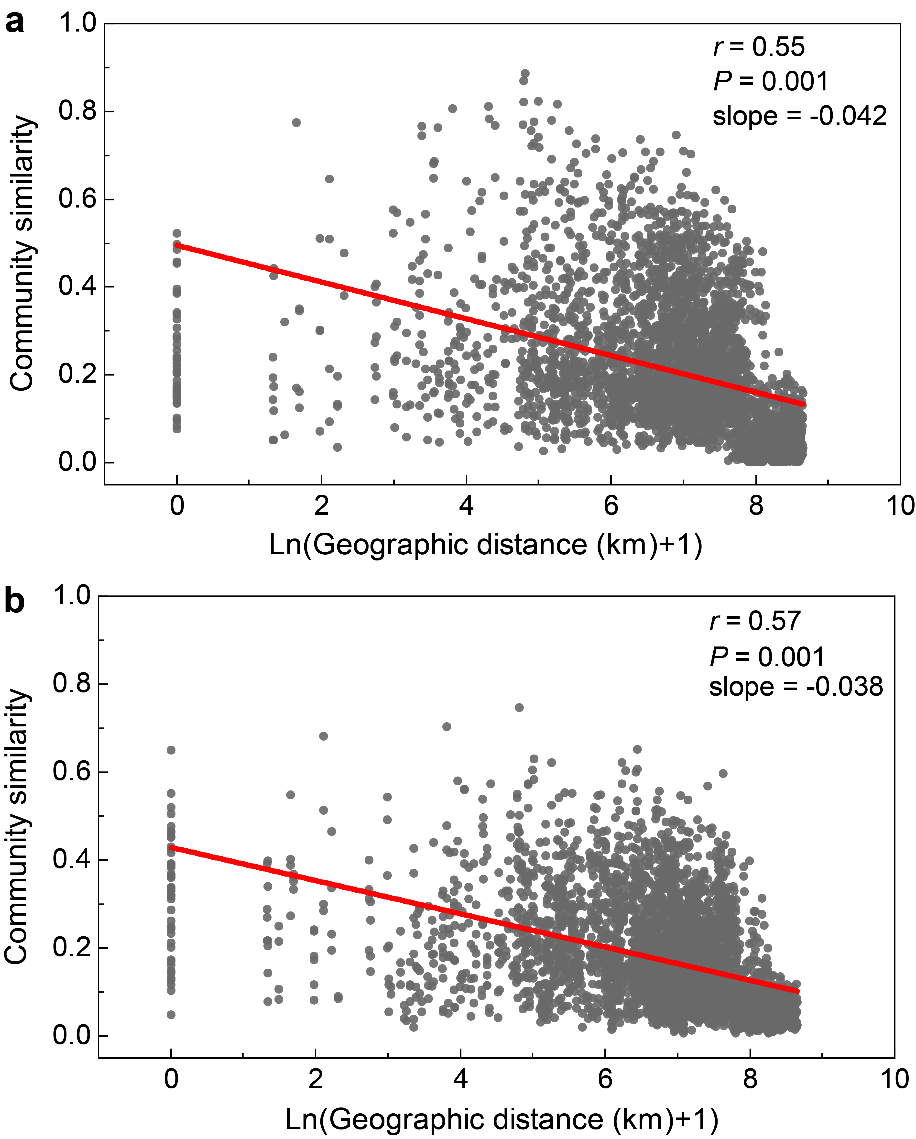


**Figure S11**. Distance-decay relationships between community similarity and geographic distance for water (**a**) and sediment (**b**) samples. Values of Mantel Spearman correlations (*r*) and probabilities (*P*) are provided. Red lines indicate ordinary least squares linear regression across all samples.


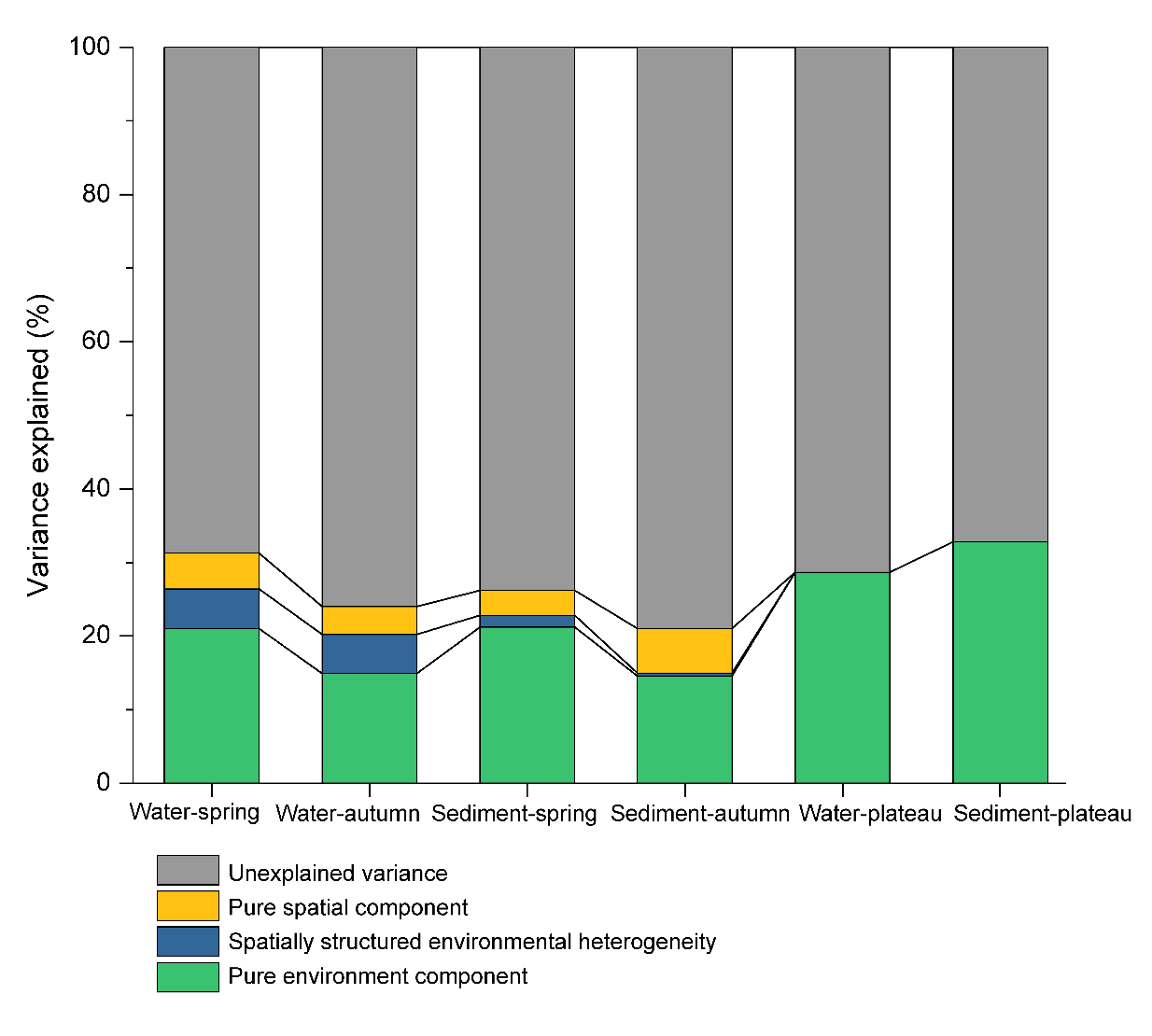


**Figure S12**. Variation in community composition explained by environmental, spatial, and spatially structured environmental components.


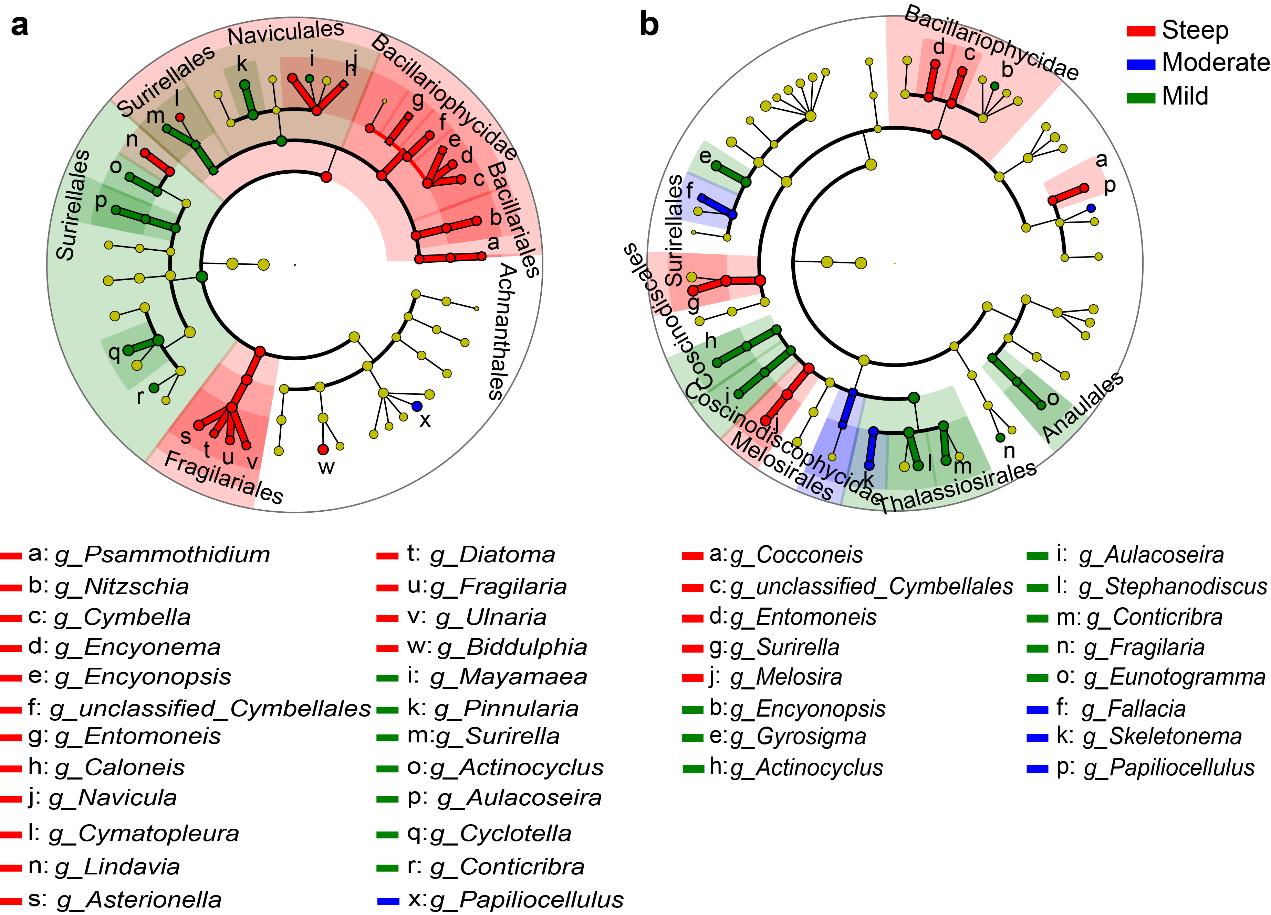


**Figure S13**. LEfSe cladogram of planktonic (**a**) and benthic (**b**) diatom communities for the three channel slope regions. Diatom taxa with a mean relative abundance of ≥ 0.1% in all samples, assigned to kingdom (innermost), phylum, class, order, family, and genus (outermost), are used to determine taxa or clades most likely to explain differences between channel slope regions. Differentially abundant taxa (biomarkers) are colored according to the slope regions in which they are most abundant; i.e. red, green and blue circles stand for biomarkers in steep, moderate and mild slope regions.


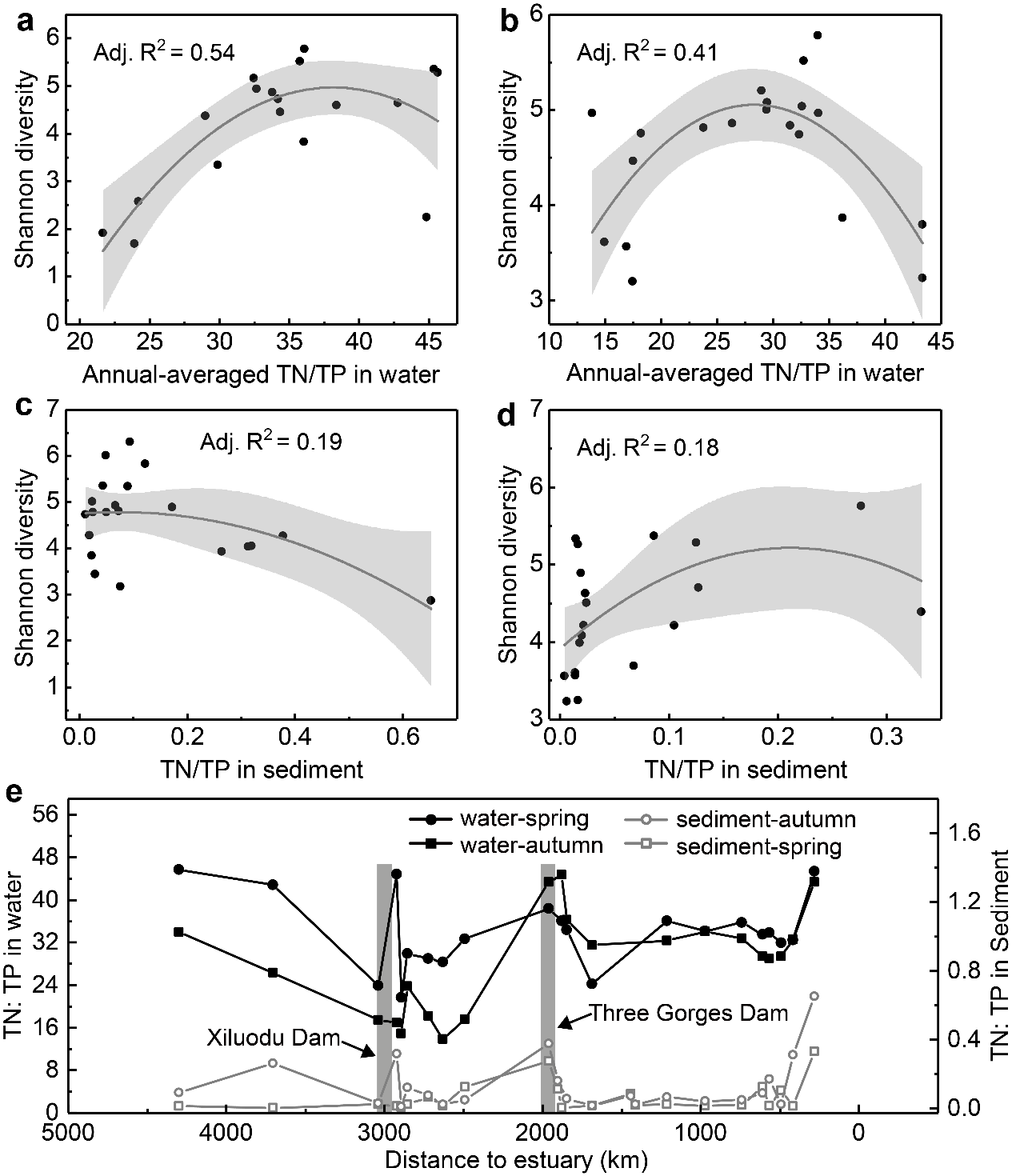


**Figure S14**. Relationships between Shannon diversity and TN:TP for water-spring (**a**), water-autumn (**b**), sediment-spring (**c**), and sediment-autumn (**d**) samples. Distance relationship of TN:TP for sampling sites along the mainstream (**e**). Annual-averaged TN:TP data during 2005−2014 are used for water samples, whereas monitored data in spring and autumn 2014 are used for sediment samples.


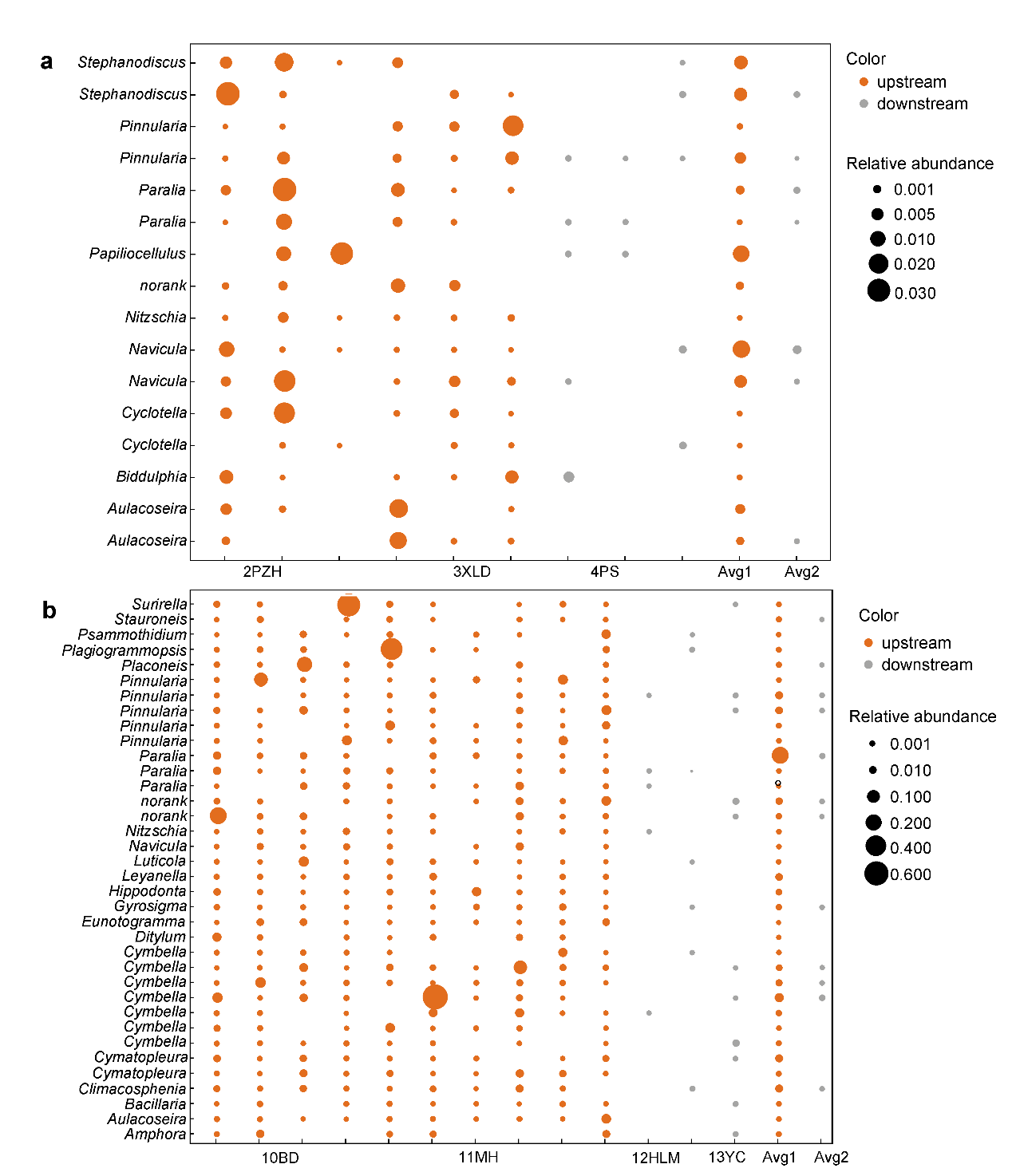


**Figure S15**. Significant differences in abundance of benthic diatoms upstream and downstream of Xiluodu Dam (**a**) and Three Gorges Dam (**b**). “Avg1” and “Avg2” respectively indicate the average abundance of benthic diatoms upstream and downstream the dams.

**Table S1**. Numbers of Indicator species and Top Indicator species across sample sites.

| Sample type | Number of indicator species* | Number of top indicator species** | Average percentage of indicator species reads in all sample reads |
| --- | --- | --- | --- |
| Sediment-plateau | 32 | 2 | 46 % |
| Water-plateau | 41 | 3 | 39 % |
| Sediment-spring | 6 | 0 | 9 % |
| Water-spring | 13 | 0 | 15 % |
| Sediment-autumn | 12 | 0 | 15 % |
| Water-autumn | 15 | 0 | 44 % |

* Including only indicator species with Indicator Value ≥ 0.3 and P-value ≤ 0.01.

** Indicator Value ≥ 0.75 and P-value ≤ 0.01.

**Table S2**. Partial Mantel test for Spearman correlations between community similarity and geographic and environmental distances.

| Correlation between diatom community similarity and: | Controlling for | Planktonic diatoms | | Benthic diatoms | |
| --- | --- | --- | --- | --- | --- |
|  |  | *r* | *P* | *r* | *P* |
| Geographic distance | Environmental distance | 0.39 | 0.001 | 0.42 | 0.001 |
| Environmental distance | Geographic distance | 0.26 | 0.001 | 0.22 | 0.001 |

**Table S3**. Effects of selected factors on diatom community using canonical correspondence analysis.

| Variables | Water-spring | | Water-autumn | Sediment-spring | Sediment-autumn | Water-plateau | Sediment-plateau |
| --- | --- | --- | --- | --- | --- | --- | --- |
|  | R^2^ | | R^2^ | R^2^ | R^2^ | R^2^ | R^2^ |
| Water Temperature | — | | 0.435** | 0.367** | 0.419*** | 0.880*** | 0.949*** |
| Ammonium Nitrogen | — | — | | — | 0.461** | — | 0.658*** |
| Chemical Oxygen Demand | 0.385*** | — | | — | — | — | — |
| Nitrate Nitrogen | 0.197* | 0.167* | | 0.229* | — | — | 0.649*** |
| PCNM-1 | 0.364*** | 0.507*** | | 0.594*** | 0.373*** | — | — |
| PCNM-2 | — | — | | — | 0.285** | — | — |
| pH | 0.175* | — | | 0.206* | 0.599** | — | — |
| Suspended Solids | 0.21* | 0.267** | | — | — | — | — |
| Total Nitrogen (TN) | 0.347*** | 0.292*** | | 0.406*** | 0.325** | — | — |
| Total Organic Carbon | — | — | | 0.274** | — | — | — |
| Total Phosphorus (TP) | — | — | | 0.334* | — | 0.979*** | — |
| TN:TP | 0.156* | 0.198* | | 0.310* | 0.245* | — | — |

Significant codes: ***≤ 0.001 **≤ 0.01 *≤ 0.05
